# Molecular-level interactions governed by temperature and composition in double salt ionic liquid-water systems

**DOI:** 10.1039/d6ra00801a

**Published:** 2026-06-04

**Authors:** K. M. Golam Rahman, Mohammad Hossain, Md. Abu Bin Hasan Susan

**Affiliations:** a Department of Chemistry, University of Dhaka Dhaka 1000 Bangladesh; b Department of Chemistry, Bangladesh University of Engineering and Technology (BUET) Dhaka 1000 Bangladesh; c Dhaka University Nanotechnology Center (DUNC), University of Dhaka Dhaka 1000 Bangladesh susan@du.ac.bd

## Abstract

An equimolar mixture of two ionic liquids (ILs), 1-butyl-3-methylimidazolium tetrafluoroborate, [C_4_mim]BF_4_ and 1-butyl-3-methylimidazolium methyl sulfate, [C_4_mim][MeSO_4_], formed a double salt ionic liquid (DSIL), [C_4_mim](BF_4_)_0.5_[MeSO_4_]_0.5_. Volumetric and viscometric analyses revealed the thermophysical and dynamic properties of ILs, DSIL, and DSIL-water binary systems throughout a considerable range of temperatures (293.15–328.15 K). The [MeSO_4_]^−^ anion engages in more pronounced directional ion–dipole and hydrogen-bond associations with the cation in comparison with the BF_4_^−^ anion. The positive excess molar volume, *V*_m_^*E*^ and the reduction in energy barrier, *E* for viscous flow, revealed that the formation of the DSIL from its parent ILs transforms a rigid, strongly correlated ionic network into a flexible, dynamically disordered structure. The decreased free energy change, Δ*G* and increased entropy change, Δ*S* unveil the coexistence of weakly coordinating BF_4_^−^ and strongly hydrogen-bonding [MeSO_4_]^−^ anions in DSIL disrupt uniform packing and enhance free volume. A non-ideal behavior of the binary system was observed across all mole fractions. A less structured form of the mixture is observed in both water-rich (*x*_DSIL_ = ∼0.1) and DSIL-rich (*x*_DSIL_ = ∼0.9) regions than in the intermediate mole fraction regions of DSIL (*x*_DSIL_ = 0.5–0.8) from the volumetric study. The dynamic viscosity (*η*) profiles, analyzed by using the Vogel–Fulcher–Tammann (VFT) model, showed a sharp decrease in *η* of pure DSIL with temperature, while the temperature sensitivity diminished upon addition of water. The *E* for viscous flow is the highest at 15.57 kJ mol^−1^ near 0.8 mole fraction of DSIL, indicating significant cluster formation. Analysis of viscosity deviation (Δ*η*) revealed negative deviations at low DSIL mole fractions, shifting to positive deviations beyond 0.8, highlighting the role of hydrogen bonding and cluster formation. Dynamic light scattering (DLS) suggested three distinct cluster sizes, which are small (0.6–1.0 nm), intermediate (1.3–68.1 nm), and large (4150.0–5560.0 nm), and the intermediate-sized mixed clusters diminish above 0.8 mole fraction. This study provides insight into DSIL-water molecular interactions, crucial for optimizing DSIL applications in green chemistry, energy storage, and drug delivery systems.

## Abstract 2

The degree of hydrogen bonding rises in the liquid structure until the mole fraction of DSIL reaches a certain value (∼ 0.8), beyond which the hydrogen bonding with water becomes weaker. Increasing temperature gives a more packed structure.

## Graphical abstract

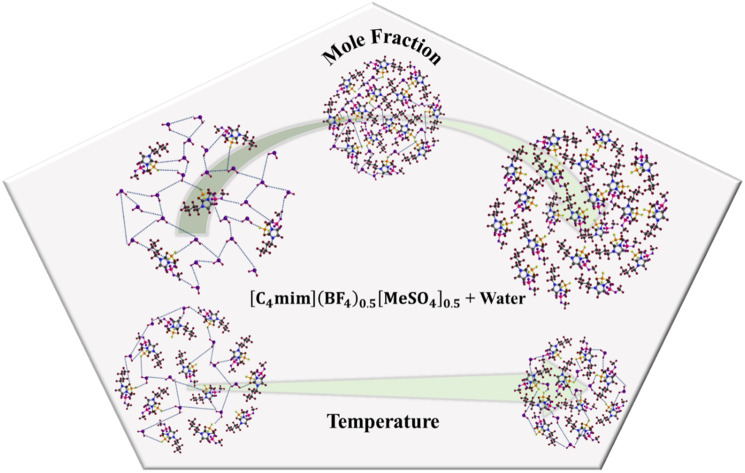

## 1. Introduction

The development of new solvents has received an upsurge of interest over the last several decades because of their vital role in expanding applications in environmental technologies, chemical synthesis, energy storage, and catalysis. Ionic liquids (ILs) have attracted special attention because of their exceptional physicochemical properties. These are liquid salts made up entirely of massive, asymmetrical cations combined with an extensive variety of inorganic and organic anions possessing melting points below 100 °C.^1–3^ The neoteric solvents are highly attractive due to their properties including minimal vapor pressure, non-flammable nature, and high ionic conductivity. Moreover, they demonstrate remarkable thermal, chemical, and electrochemical robustness.^4^ These exceptional characteristics have enabled ILs to serve as electrolytes in a variety of energy devices. For instances, they are used in lithium-ion secondary batteries, electrical double-layer capacitors, and supercapacitors. ILs find applications in fuel cells, solar cells, and actuators. They are also employed as media for electrodeposition.^5–8^ Notwithstanding their many benefits, ILs have drawbacks in this specific area. For instance, the intrinsically high density (*ρ*), dynamic viscosity (*η*), and markedly poor conductivity (*κ*) of pure ILs limit their suitability as electrolytes in commercial electrochemical systems. Furthermore, insufficient interfacial contact between the solute and IL medium causes delayed and ineffective processes like cellulose dissolution.^9–12^

In order to overcome these constraints, double salt ionic liquids (DSILs), combinations of two or more ILs, were introduced.^13^ Chatel *et al.* raised an important question about the formation of mixed ILs: are they simply IL mixtures or double salts? The investigation demonstrated the necessity of introducing the idea of DSILs for ILs that are made up of more than two different types of ILs.^13^ Since each ion combination should be regarded as a distinct IL under this framework, mixed ILs are actually new ILs made up of multiple types of anions and/or cations, or DSILs, rather than just mixtures of ILs. DSILs can consist of several cations and anions, one cation and multiple anions, or one anion and multiple cations.^13–15^ The individual constituents of distinct ILs cannot be identified separately. Simply put, the chemistry is based on the ions that make them up and how each ion interacts with the others, regardless of the counter-ion. The distinct ionic environments that DSILs provide improved tunability of important characteristics like solvation ability, *κ*, and *η*. DSILs have outperformed their parent ILs in terms of reduced *η* and increased conductivity by customizing their ionic compositions. This makes DSILs particularly interesting for CO_2_ capture, electrocatalysis, advanced batteries and fuel cells, chemical separation procedures, and green synthesis techniques, among other next-generation energy devices.^16–19^ Another significant application of the ILs and DSILs system is in synthesis of nanomaterial and control of morphology. The hydrophilic imidazolium ILs, including [C_2_mim](CH_3_SO_4_), [C_4_mim](CH_3_SO_4_), and [C_2_mim](C_2_H_5_SO_4_), have been demonstrated to be effective soft templates and self-directed molecules for the hydrothermal synthesis of ZnO nanoparticles with controlled flower, petal, and flake structures.^20^ Given their more complicated ionic environment, it is expected that the use of DSILs will allow for better control of formation of nanoscale structure and self-assembly processes compared to regular ILs or simple IL mixtures. The term DSIL as used here conforms to the existing literature in which DSIL refers to an ionic mixture containing at least two different ionic compounds within the same ionic matrix.^13^ These substances do not indicate the synthesis of any new stoichiometric compound; instead, they involve ions that interact with each other dynamically and have a statistical distribution. Therefore, DSILs can be considered complex ionic fluids with heterogeneity in their local structures and not distinct molecules. This viewpoint fits into theoretical frameworks like pseudo-lattice and dynamic networks, where the ionic interactions are temporary and result in local non-homogeneity of the environment.^13^ Tunability of physical and chemical properties constitutes one of the important advantages of DSILs. Depending on the type and proportion of the constituent ions, the DSILs can show properties which could be somewhere between or even better than those of the ILs. However, despite all the developments in the field, there is still a scientific issue that needs to be addressed about ILs. For instance, when two different ILs are mixed within a similar ionic structure, will they only be a physical mixture or something different, such as DSILs, with emerging supramolecular attributes? The solution to this problem is based on knowing how ion distribution, mixed anions, and local structure variations affect intermolecular interactions and macroscopic physicochemical properties. In this study, the first step involves examining whether the equimolar mixture of [C_4_mim]BF_4_ and [C_4_mim][MeSO_4_] causes simple mixing or formation of a new DSIL structure. Special attention has been paid to studying how the presence of mixed anions affects ion association, hydrogen bonding, coulombic forces, and mesoscopic aggregation in comparison with parent ILs.

Another crucial method to get over the drawbacks of ILs is to combine them with conventional molecular liquids. The *ρ*, *η*, *κ*, and polarity of ILs can all be considerably changed by water and other polar solvents.^21^ Furthermore, ILs are hygroscopic substances; even hydrophobic ILs have been shown to take some water from the atmosphere.^22^ ILs and water serve as functional fluids in bioscience when they are deliberately mixed.^23–25^ Hemicellulose, a source of raw materials for many high-value products, can be dissolved with the help of IL-water binary systems.^26^ For applications like friction, lubrication, or catalysis, where the interfacial stacking of ILs is a crucial component to customize functioning, water present in IL charges the surface. Absorption refrigeration technology may benefit from the use of IL aqueous solutions.^26^ Combining these strategies, recent work has begun to explore DSIL-water binary mixtures, which add another layer of tunability. These complex systems offer the possibility to fine-tune supramolecular interactions. Nevertheless, systematic research is necessary to fully unleash the potential of these interactions in DSIL-water systems, as their molecular-level knowledge is still unexplored.

In this context, the current investigation not only focuses on the DSIL self-assembly but goes a step further by investigating the effect of water content and temperature on the supramolecular structure and thermodynamics of DSIL-water binary solutions. This study is conducted at the interface of solution thermodynamics and fluid heterogeneity. In particular, this study seeks to understand how hydration affects hydrogen bonding, ion–dipole interaction, coulombic attraction, and aggregation in mixed anions ionic liquids, and how these molecular level changes manifest in observable macroscopic properties such as density, viscosity, and aggregation.

While individual ILs along with solvent molecules have been widely studied,^14,31,32^ a thorough investigation of the molecular interplays, behavior of cluster formation, and thermodynamic properties of DSIL-water binary systems remains limited. There is a particular lack of data on how water modulates the structural organization and dynamics within DSIL environments. This study builds on the previous research by conducting an extensive investigation of temperature-dependent behavior,^27–30^ which provides insight into the thermal properties of the system.^31,32^ In addition, different analytical methods, such as volumetric analysis, viscometry, dynamic light scattering (DLS), also named as photon correlation spectroscopy (PCS), and near-infrared (NIR) spectroscopy, were used to characterize the properties of the system at various levels. This multidisciplinary strategy enables the provision of novel information regarding the clustering behavior and hydrogen bonding dynamics in DSIL-water systems, which are not well understood in existing studies. Nevertheless, additional validation through sophisticated spectroscopic methods (such as NMR and DSC) will prove invaluable in future endeavors. Previous studies on binary ionic liquid systems, such as mixtures of [dema]HSO_4_ and [dema][NTf_2_], have demonstrated that techniques such as FT-IR, ^1^H NMR, and DSC can effectively probe the formation of DSIL through the observation of dynamic ion exchange, hydrogen-bond redistribution, cooperative mixed-ion environments, and composition-dependent thermal behavior.^33^

This study aims to bridge the knowledge gap by systematically investigating the supramolecular interactions, thermodynamic behavior, and cluster formation in DSIL-water binary mixtures over varying compositions and temperatures. The DSIL investigated is a 1 : 1 molar mixture of two imidazolium-based ILs: 1-butyl-3-methylimidazolium tetrafluoroborate, [C_4_mim]BF_4_, and 1-butyl-3-methylimidazolium methyl sulfate, [C_4_mim][MeSO_4_]. The resulting DSIL is denoted as [C_4_mim](BF_4_)_0.5_[MeSO_4_]_0.5_, representing a unique system with balanced contributions from both BF_4_^−^ and [MeSO_4_]^−^ anions. The study investigates the physicochemical properties and molecular-level interactions of this DSIL in binary mixtures with water across a range of DSIL mole fractions and temperatures.

This investigation represents the first report of temperature and composition-dependent supramolecular interactions in [C_4_mim](BF_4_)_0.5_[MeSO_4_]_0.5_-water systems by the volumetric, viscometric, photon correlation, and NIR spectroscopic investigations. The volumetric study unveils the coulombic interactions, dipolar interactions, induced dipole effects, dispersion forces, and hydrogen bonds present in the system. The weakening of water self-association for the disruption of hydrogen bonds and reduction in cation–anion interactions within DSIL for coulombic and van der Waals forces have been investigated. Ion-polar group association is observed in the binary systems. Viscometric studies have been applied to qualitatively assess the extent of intermolecular forces considering the molecular dimensions and geometry of the components in the binary systems. Interactions between unlike species, such as hydrogen bonding and the development of charge-transfer associations, are also detected. A comparison of the sensitivity of temperature and composition is also inferred by this method. Photon correlation spectroscopy has been employed to examine the size of aggregates and the structural organization in DSIL-water binary mixtures. NIR spectroscopy has been used to investigate the influence of temperature and composition on hydrogen bonding, as reflected by the population of free –OH groups. The structure of water has been explained by a quasi-two-state model from the 2D correlation diagram of NIR spectroscopy. These thorough studies offer important new information about the structure–property relationships controlling DSIL-water binary mixtures, demonstrating how composition and temperature affect fluidity, hydrogen bonding dynamics, and supramolecular organization.

## 2. Methods and instrumentation

**Scheme 1:**
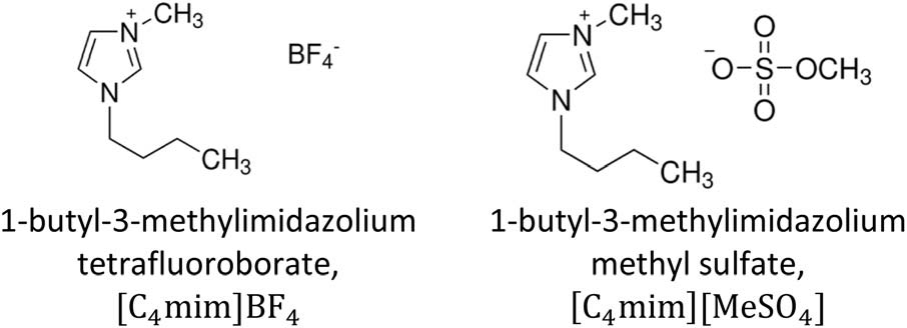
Chemical structures of pure ILs, [C_4_mim]BF_4_ and [C_4_mim][MeSO_4_].

The ILs, 1-butyl-3-methylimidazolium tetrafluoroborate, [C_4_mim]BF_4_, and 1-butyl-3-methylimidazolium methyl sulfate, [C_4_mim][MeSO_4_], were procured from Sigma Aldrich (≥98.0%, HPLC) and utilized without further treatment (Scheme 1). Ultrapure water with a specific conductance of 0.055 µS cm^−1^ prepared using BOECOpure (Model No. BOE 8082060, Germany) was used for preparing binary mixtures, cleaning, and analysis. To prepare the DSIL, [C_4_mim](BF_4_)_0.5_[MeSO_4_]_0.5_, both ILs were simply mixed, keeping the mole fraction of each one at 0.5. Water was added to DSIL to prepare the binary mixtures. The DSIL-water binary mixtures were prepared at intervals of 0.1 mole fraction from 0.9 to 0.1. Following preparation, the DSIL and the DSIL-water mixtures were sonicated for 60 min using a sonicator (LU-2 Ultrasonic Cleaner, Labnics, USA) to ensure homogeneous mixing. A DMA 4500 M (Anton Paar, UK) density meter measured the *ρ* of the systems with a repeatability of 10^−5^ g cm^−3^ and an accuracy of 5 × 10^−6^ g cm^−3^. The viscosities were measured using a microviscometer (Lovis 2000 ME, Anton Paar, UK) by the rolling ball principle with an accuracy of ±10^−6^ mPa s. Glass capillaries of 1.59 mm and 2.50 mm in diameter, together with gold balls, were employed for the measurements. To control the temperature, an integrated temperature controller with a precision level of ±0.01 K was applied. An automated refractometer (Anton Paar, Model: Abbemat 300) was used to monitor the refractive index. There was a resolution of ±1 × 10^−5^ in the measurements. An integrated Peltier thermostat was used for temperature monitoring. The incident sodium D line yellow light had *λ* = 589.3 nm. The refractometer was validated with deionized water and a built-in software function referred to as “Set Check” with a tolerance of ±1 × 10^−4^, and a reference value of 1.3330. Hydrodynamic diameters (*D*_h_) of the aqueous solutions were monitored using the Zetasizer Nano ZS90 (ZEN3690, Malvern Instruments Ltd, UK). Polystyrene latex was used as a standard for instrument validation. To hold the samples, a glass cell was utilized. With a scattering angle of 90°, a He–Ne laser beam (632.8 nm) served as the light source. Cluster sizes were evaluated based on intensity measurements. To make sure that the process is reliable and reduce any possibility of dust interference or other kinds of particles, all samples were first filtered through a 0.45 µm polytetrafluoroethylene membrane filter before analysis. Following the filtration process, the samples were left to stand for approximately 24 hours to allow them to equilibrate before DLS measurement was carried out. In addition, every measurement was repeated three times, and consistent results were obtained. Inbuilt thermostats were used to control the temperature of the instrument, which was maintained within ±0.01 K of the desired level. Utilizing a Fourier transform infra-red/near infra-red (FT-IR/NIR) spectrophotometer (PerkinElmer, USA), NIR spectra were recorded. In order to retain a resolution of 4 cm^−1^, 32 scans were employed for each measurement, spanning the wavenumber 4000 cm^−1^ to 10 000 cm^−1^. It was validated with a polystyrene film. CaF_2_ windows (Specac Model No.: GS20730) with 0.1 mm spacing were employed to sample the solutions. To regulate the experimental temperatures, an electrical heating jacket (Specac Model No.: GS20730) was utilized. To prevent compositional variations arising from the vaporization of water, the *ρ* of the mixtures were not measured at temperatures higher than 328.15 K. Below this temperature, the vapor pressure of water remains sufficiently low (less than 0.031 MPa), rendering any compositional changes negligible. Nonetheless, measurements were extended up to this upper limit to examine whether any anomalous structural behavior of water occurs near 50 °C.^34^

## 3. Results and discussion

### 3.1. Double salt ionic liquid from the combination of pure ionic liquids

A linear decrease in the *ρ* of pure ILs, [C_4_mim]BF_4_ and [C_4_mim][MeSO_4_] and the DSIL, [C_4_mim](BF_4_)_0.5_[MeSO_4_]_0.5_ with increasing temperature is observed (*cf.* Fig. S1, SI). The significant decrease indicates the weakening of electrostatic, dipolar, polarization-induced, dispersive, and hydrogen-bonding forces.^35^

The excess molar volume (*V*_m_^*E*^) is a key thermodynamic parameter that represents the discrepancy in molar volume between the experimental binary system and its ideal counterpart.^36^ It is a complex property that is affected not only by the molecular dimensions, geometry, and chemical characteristics of the constituents of a mixture^37,38^ but also by the intra- and intermolecular interactions involving solute and solvent molecules and conformational influences caused by the disparities in molar and free volumes among the constituents of the solution.^39^ Therefore, to investigate the deviation from ideal additivity behavior for DSIL, the *V*_m_^*E*^ was calculated using,1
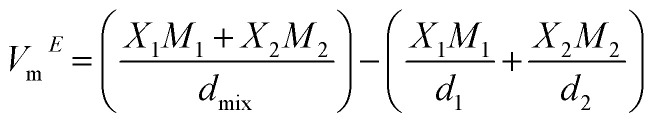
where *d*_1_ and *M*_1_ are the density and molar mass of [C_4_mim]BF_4_; *d*_2_ and *M*_2_ are the density and molar mass of [C_4_mim][MeSO_4_].

**Fig. 1:**
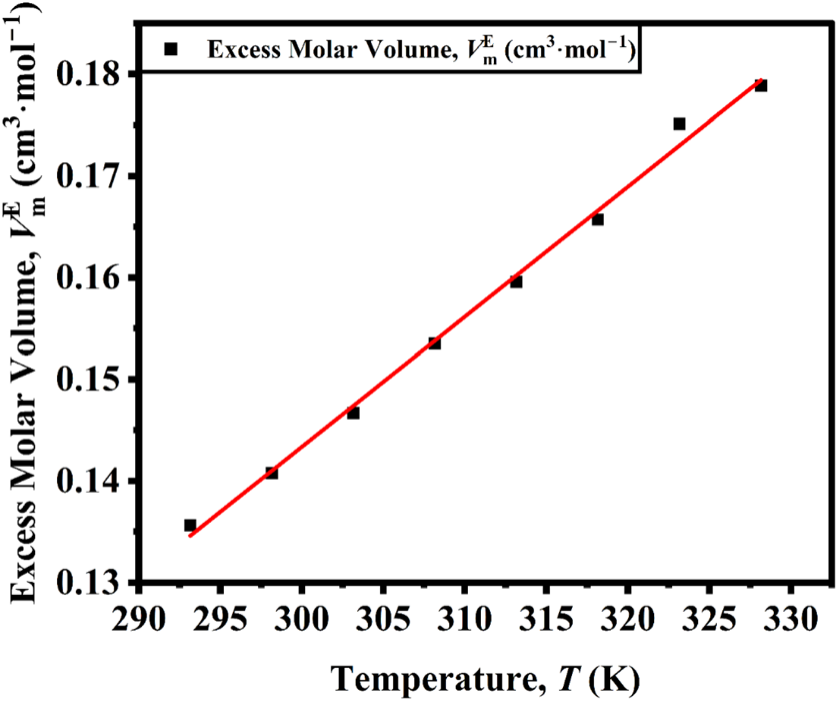
Excess molar volume of [C_4_mim](BF_4_)_0.5_[MeSO_4_]_0.5_ as a function of temperature.

A linear increase in positive *V*_m_^*E*^ observed in Fig. 1 indicates progressive expansion of the liquid structure of DSIL. When [C_4_mim]BF_4_ and [C_4_mim][MeSO_4_] are mixed, the differing anion sizes, shapes, and interaction strengths between BF_4_^−^ and [MeSO_4_]^−^ hinder the efficient packing of ions that occurs in the pure ILs. As BF_4_^−^ is a relatively small, symmetrical, weakly coordinating anion and [MeSO_4_]^−^ is larger, more asymmetric, and has stronger hydrogen-bonding capability due to its polar –SO_3_ group, their coexistence disrupts the uniform cation–anion network, introducing voids or free volume in the liquid structure. In pure ILs, the strong coulombic attractions between [C_4_mim]^+^ and its counterion leads to compact structures. Upon mixing, the interaction strength between the cation and the two distinct anions becomes uneven, leading to partial weakening of the electrostatic attraction. The temperature-dependent linear increase of *V*_m_^*E*^ also suggests that the thermal agitation enhances molecular motion and further disrupts the structure of DSIL.

A pronounced reduction in the *η* of both pure ILs and DSIL is observed as the temperature increases. For each system, the Vogel–Fulcher–Tammann (VFT) model was employed to correlate the experimental *η* values, which accurately represent the dependence of *η* on temperature at a fixed molar composition. For comparison, the viscosity data were also fitted using the Arrhenius equation (ln(*η*) = ln *A* + *E*_a_/*R*·1/*T*; where *A* and *E*_a_ are fitting coefficients), which showed good agreement over the studied temperature range (*R*^2^ ≈ 0.997–0.998) (*cf.* Fig. S2, SI and *cf.* Table S1, SI). But the VFT model showed excellent agreement (*R*^2^ ≥ 0.999) (*cf.* Fig. S3, SI and *cf.* Table S2, SI), and it also considered the small non-linear effect of the temperature on the transport parameters, hence it was used in subsequent studies. Moreover, ILs have been demonstrated to display non-Arrhenius transport characteristics owing to their heterogeneous nature.^40–44^ As a result, the VFT model, which considers this type of complexity, is better suited to represent viscosity in such systems. The VFT model is as follows.2
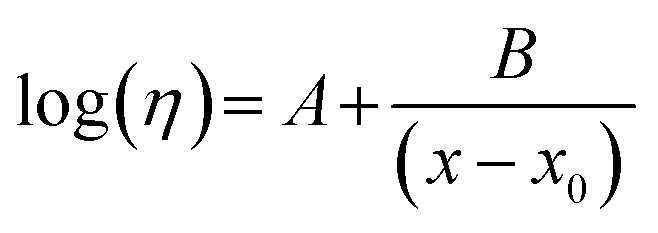
where *η* stands for the viscosities of mixtures at constant mole fraction, *A*, *B*, and *x*_0_ are the fitting coefficients and *x* is the absolute temperature in Kelvin.

The viscosities followed VFT behavior (*cf.* Fig. S3, SI). [C_4_mim][MeSO_4_] displays the highest *η*, reflecting its larger, more polar anion ([MeSO_4_]^−^) that forms stronger directional ion–dipole and hydrogen bonds with the cation. By contrast, the DSIL shows the minimum *η*. The reduction in *η* of the DSIL in comparison with its parent ILs results from the disruption of homogeneous ionic networks. The coexistence of dissimilar anions (BF_4_^−^ and [MeSO_4_]^−^) hinders efficient packing and coordination with the cation, introducing structural disorder and additional free volume that eases ion mobility. This mixed-anion environment also weakens long-range ion correlations and reduces the overall cation–anion interaction strength, leading to less cooperative dynamics. Consequently, the VFT parameters (*B* and *x*_0_) decrease, indicating a smaller activation barrier and a more dynamically flexible structure. Thus, the DSIL replaces the strong, directional cohesion of the pure ILs with a heterogeneous, loosely packed framework, yielding enhanced fluidity and lower *η*.

The viscosity deviation (Δ*η*) represents the discrepancy between the experimentally measured *η* and that predicted for an ideal binary mixture.

3 Δ*η* = *η*_experimental_ − [*x*_1_*η*_1_ + *x*_2_*η*_2_]

The symbols *η* and *x* correspond to viscosity, measured in mPa s, and mole fraction, respectively. The subscript 1 corresponds to [C_4_mim]BF_4_ and the subscript 2 corresponds to [C_4_mim][MeSO_4_], respectively.

**Fig. 2:**
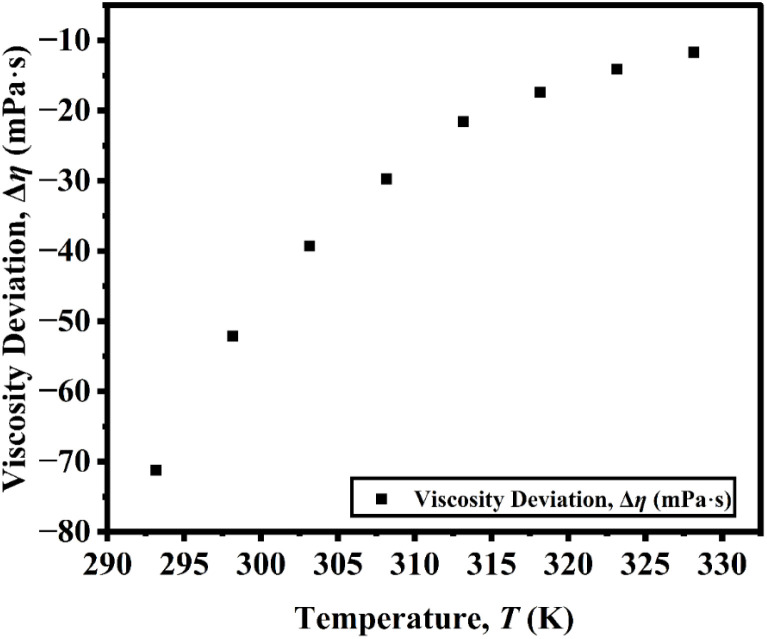
Viscosity deviation of [C_4_mim](BF_4_)_0.5_[MeSO_4_]_0.5_ as a function of temperature.

The Δ*η* of the DSIL increases with temperature and gradually approaches a limiting value (Fig. 2), indicating temperature-induced modification in the ionic microstructure. At lower temperatures, strong coulombic interactions and hydrogen-bonding interactions between the cations and anions of the parent ILs promote the formation of well-organized ion networks, resulting in large structural heterogeneity and a more negative Δ*η* due to stronger cohesive forces relative to the ideal mixture. As temperature increases, thermal motion progressively weakens these directional interactions, leading to partial disruption of ion pairs and increased free volume.

Consequently, the Δ*η* becomes less negative and eventually stabilizes at higher temperatures when the system reaches a dynamically equilibrated state with reduced ion–ion correlation.

The energy barrier (*E*) for individual ILs and the DSIL at different temperatures was computed from eqn (4) (ref. 45) and is illustrated in Fig. S4 (SI).4
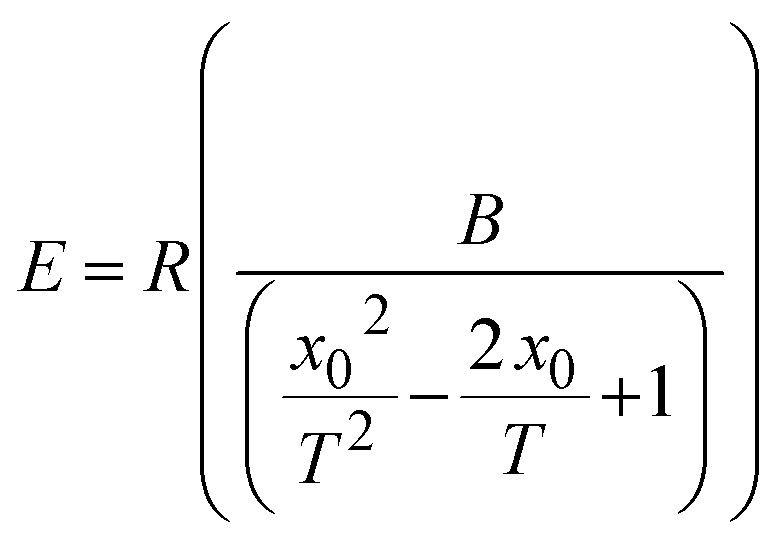
where *R* is the molar gas constant (8.3145 J K^−1^ mol^−1^), *B* and *x*_0_ are the fitting coefficients of the VFT equation.

In all cases, *E* shows a gradual decline as the temperature rises, reflecting the temperature-induced reduction in cohesive interactions and enhancement in ion mobility. However, the DSIL exhibits comparatively lower *E* values throughout the studied temperature range, suggesting that the mixed-anion environment facilitates easier ionic rearrangement and reduces the energy of viscous flow. The lower values of *E* for DSIL compared to the parent ILs further indicate a more flexible and dynamically equilibrated ionic structure, where the competing electrostatic and dispersive interactions between BF_4_^−^ and [MeSO_4_]^−^ anions mitigate strong directional forces. This cooperative anionic environment leads to improved structural homogeneity and lower resistance to ion motion, thus accounting for its reduced *E*.

The Gibbs free energy of activation (Δ*G*) for viscous flow in both the pure ILs and the DSIL was determined using the Eyring equation.5
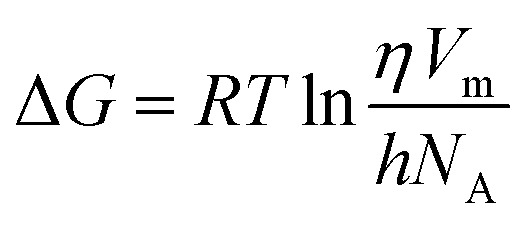
where *η* denotes the dynamic viscosity of the liquid (Pa s); *h* represents Planck constant; *N*_A_ stands for Avogadro number; *V*_m_ indicates the molar volume of the liquid (m^3^ mol^−1^); and *T* corresponds to the absolute temperature.

**Fig. 3:**
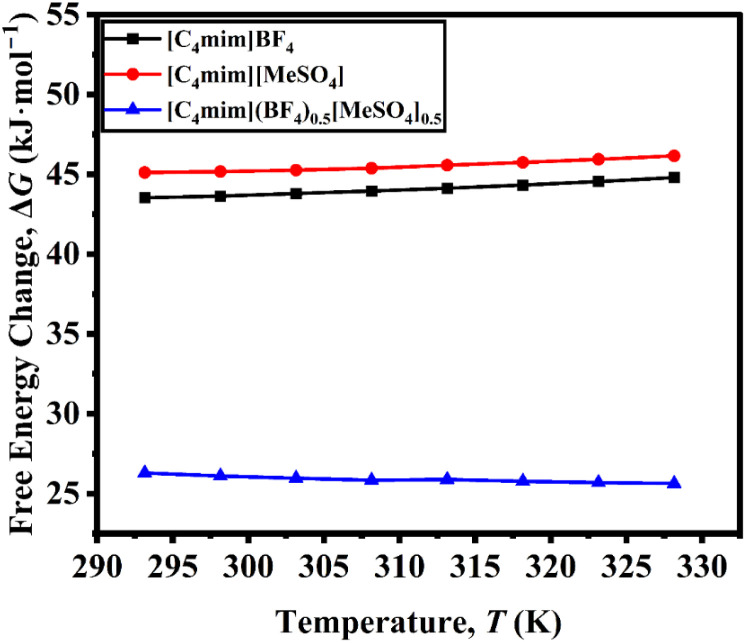
Changes in free energy for the viscous flow of [C_4_mim]BF_4_, [C_4_mim][MeSO_4_], and [C_4_mim](BF_4_)_0.5_[MeSO_4_]_0.5_ as a function of temperature.

The Δ*G* represents the minimum energy required for ions to overcome the potential barrier for viscous flow and thus reflects the extent of structural ordering and ion–ion interactions within the liquid. For both pure ILs, Δ*G* increases almost linearly with temperature (Fig. 3), indicating that viscous flow becomes progressively less favorable as thermal agitation disrupts the local cation–anion coordination environment and increases the energy needed to reorganize the liquid structure. Similar temperature dependence has been reported for imidazolium-based ILs, where stronger ion pairing and structured hydrogen-bonding networks lead to higher Δ*G* values at elevated temperatures. In contrast, the DSIL exhibits a distinctly lower Δ*G* and a decreasing trend with increasing temperature (Fig. 3). This suggests that the mixed-anion system possesses a more dynamically disordered and less cohesive structure than the pure ILs. The presence of two chemically distinct anions (BF_4_^−^ and [MeSO_4_]^−^) introduces structural incompatibility and heterogeneity, weakening the directional ion–ion interactions and reducing the *E* for structural rearrangement. Consequently, the DSIL requires less activation energy for viscous flow, implying a more facile molecular motion and reduced internal resistance. The declining Δ*G* with temperature further supports that thermal energy promotes increased molecular freedom and reduced cooperative interactions within the mixed-anion matrix.

The change in entropy of activation for viscous flow, Δ*S* was calculated using the derivative of free energy change, −d(Δ*G*)/d*T* from Fig. 3. For all systems, Δ*S* decreased with rising temperature (*cf.* Fig. S5, SI), implying that thermal excitation promotes structural ordering and reduces configurational freedom during viscous flow. However, a distinct difference is observed between the pure and mixed systems: while both pure ILs exhibit negative Δ*S* values over the studied range (293.15–328.15 K), the DSIL displays positive Δ*S* values with a slight depression at intermediate temperatures (308.15 K and 313.15 K). The negative Δ*S* for the individual ILs indicates a more organized transition state relative to the initial liquid state, consistent with strong cation–anion correlations and restricted molecular rearrangement. In contrast, the positive Δ*S* for the DSIL suggests enhanced disorder and reduced ion–ion coupling in the activated state, reflecting a higher degree of molecular mobility and weaker structural cooperativity. The temporary lowering of Δ*S* around 308–313 K may be attributed to subtle structural reorganization or competition between the two anions (BF_4_^−^ and [MeSO_4_]^−^), leading to transiently increased packing efficiency and reduced configurational entropy.

The enthalpy of activation (Δ*H*) associated with viscous flow was determined using

6 Δ*H* = Δ*G* + *T*Δ*S*

The Δ*H* for viscous flow shows positive values for all systems and decreases with temperature (*cf.* Fig. S6, SI), indicating that the viscous flow process is endothermic and becomes energetically less demanding at elevated temperatures. The higher Δ*H* values observed for the pure ILs compared to the DSIL reveal that the pure systems require greater energy to disrupt their well-defined ionic networks during flow. In contrast, the DSIL exhibits a lower Δ*H* throughout the temperature range, confirming the presence of weaker cohesive forces and a more dynamically labile structure due to mixed-anion coordination. The minor lowering of Δ*H* at 308.15 K and 313.15 K corresponds well with the entropy minima, supporting the notion of local structural rearrangement or reduced cooperative motion at these intermediate temperatures. Overall, the combination of positive Δ*S* and smaller Δ*H* values for the DSIL signifies a less ordered, more fluidic system with reduced activation barriers for viscous flow.

The temperature-dependent DLS pattern distinctly distinguishes the parent ILs from the DSIL and suggests the formation of a novel restructured material rather than a physical mixture. Pure [C_4_mim]BF_4_ demonstrates an increment in the particle size continuously and gradually from ∼142 nm at 293.15 K to ∼955 nm at 333.15 K, suggesting clustering due to increasing thermal energy and ions aggregation (*cf.* Fig. S7, SI). On the other hand, the pure [C_4_mim][MeSO_4_] sample displays a highly erratic aggregation process, with very small-sized clusters (∼1 nm) formed at lower temperatures, then abruptly shifting to very large aggregate sizes (4000–5000 nm) at higher temperatures (*cf.* Fig. S8, SI). This is attributed to the existence of intense local interactions in the pure sulfate system. The DSIL, [C_4_mim](BF_4_)_0.5_[MeSO_4_]_0.5_, shows a completely different trend (*cf.* Fig. S9, SI). The increase in aggregate size with increasing temperature follows a gradual trend just like [C_4_mim]BF_4_, but with much wider distribution compared to that of [C_4_mim]BF_4_. Unlike the [C_4_mim][MeSO_4_], no large aggregates form abruptly, and also unlike [C_4_mim]BF_4_, its distribution curve is not narrow. Such a combination of intermediate behavior with uniqueness suggests that the structure is not a straightforward linear superposition of the two ILs. Rather, the dual presence of BF_4_^−^ and [MeSO_4_]^−^ ions gives rise to competing and cooperating ion–ion interactions, which cause a new ionic network to form. The wide distribution of sizes and the gradual growth observed in the double salt system indicate increased heterogeneity in structure due to the mixed anions, where coulombic forces, hydrogen bonds, and steric hindrance are reorganized. The absence of extremely strong association in [C_4_mim][MeSO_4_] and the departure from uniform growth behavior in [C_4_mim]BF_4_ indicate the formation of new intermolecular interactions within the mixed system. Consequently, the DLS studies have revealed significant evidence for the formation of a genuine DSIL having specific physicochemical characteristics and not just a simple physical mixture of the two ILs.

**Fig. 4:**
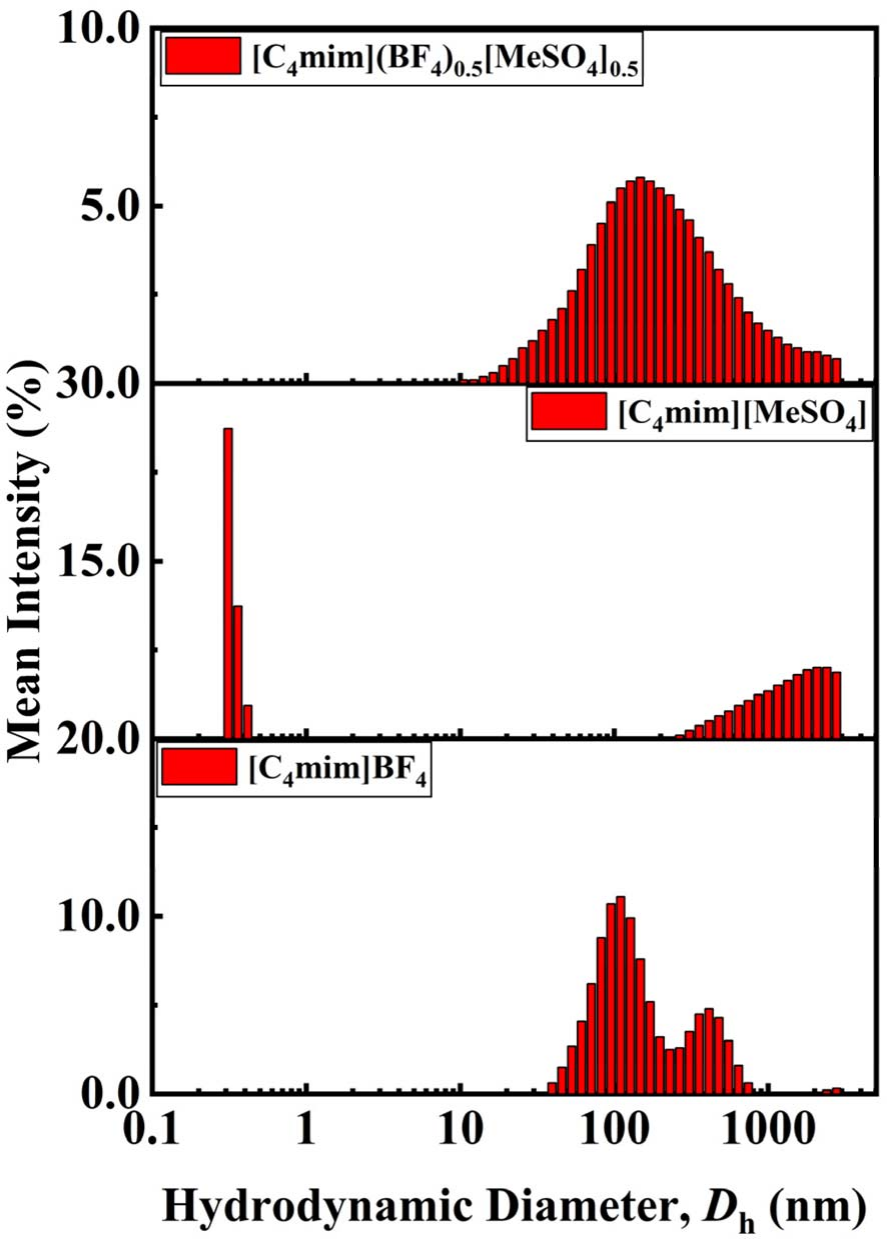
Size of aggregates formed in [C_4_mim]BF_4_, [C_4_mim][MeSO_4_], and [C_4_mim](BF_4_)_0.5_[MeSO_4_]_0.5_.

From the comparative graph of the DLS results for mean intensity against hydrodynamic diameter, it is evident that the DSIL, [C_4_mim](BF_4_)_0.5_[MeSO_4_]_0.5_, has different aggregation properties from the parent ILs, thus proving its formation into a new system and not a mere mixture (Fig. 4). For pure [C_4_mim]BF_4_, there is a relatively small size distribution within the ∼100–300 nm region, with an additional smaller peak for larger clusters, suggesting that the aggregation of this compound is quite stable and consistent. On the other hand, for pure [C_4_mim][MeSO_4_], there is a bimodal and very heterogeneous size distribution, ranging from very small clusters (<1 nm) to very large aggregates (>1000 nm). DSIL, on the other hand, shows a wide but unimodal distribution in the range of 100–1000 nm without any of the very small clusters seen in [C_4_mim][MeSO_4_] or the narrow distribution of [C_4_mim]BF_4_. The presence of this intermediate and unique aggregation pattern implies that there is a rearrangement of the ionic interactions within the DSIL system because of the simultaneous presence of BF_4_^−^ and [MeSO_4_]^−^ ions. The softening of the abrupt aggregation pattern and the presence of a new particle size distribution show that a reorganized ionic network exists within the DSIL system.

**Fig. 5:**
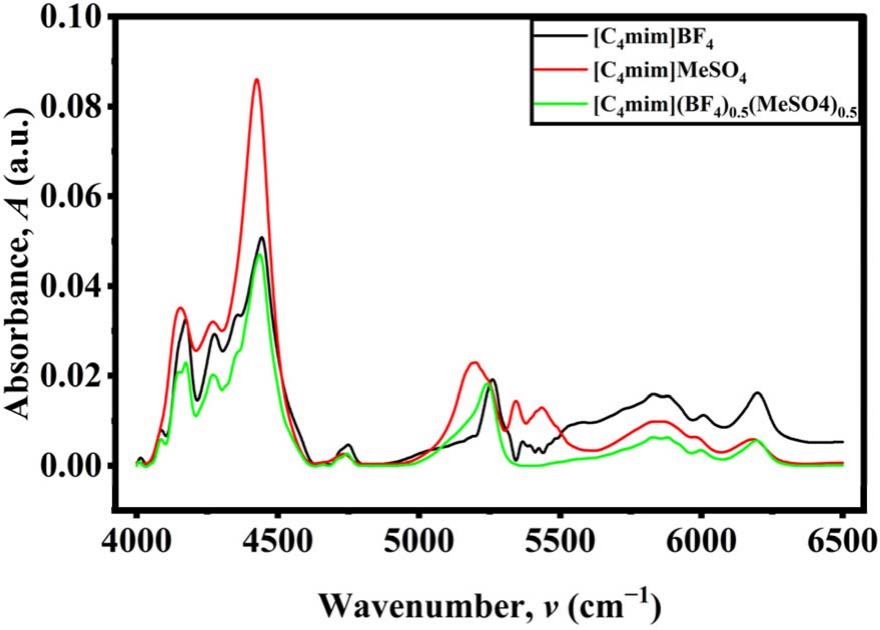
NIR spectra of [C_4_mim]BF_4_, [C_4_mim][MeSO_4_], and [C_4_mim](BF_4_)_0.5_[MeSO_4_]_0.5_ at 308.15 K from 4000 cm^−1^ to 6500 cm^−1^.

The NIR spectra of [C_4_mim]BF_4_, [C_4_mim][MeSO_4_], and [C_4_mim](BF_4_)_0.5_[MeSO_4_]_0.5_ exhibited distinctive peaks in the region of 4000 cm^−1^ to 8000 cm^−1^ (*cf.* Fig. S10–S12, SI). The bands observed in the NIR spectrum of [C_4_mim]BF_4_ (Fig. S10) at 4200–4500 cm^−1^ are due to the combination of bending and stretching vibrations of the C–H bond. On the other hand, the peaks observed in the range of 5000–6200 cm^−1^ are related to the overtones of C–H bond stretching vibrations. The weaker absorption bands occurring between 6500 and 7500 cm^−1^ result from higher combination bands of ring C–H with some contribution from BF_4_^−^ interaction. The weaker absorption bands occurring beyond 8000 cm^−1^ correspond to second overtones of C–H stretching vibrations. The lack of any significant spectral variations with respect to temperature suggests the structural stability of the IL. The prominent band observed at 4300–4400 cm^−1^ for the spectrum of [C_4_mim][MeSO_4_] (*cf.* Fig. S11, SI) has been assigned to be the result of imidazolium ring C–H and aliphatic C–H stretches with O–S–O bends. Bands found in the 5200–6200 cm^−1^ region represent the first overtones of aliphatic and aromatic C–H stretching. The NIR spectrum of [C_4_mim](BF_4_)_0.5_[MeSO_4_]_0.5_ is characterized by the combination and overtone bands of the imidazolium cation, proving that both parent ILs have been incorporated into a single molecular framework (*cf.* Fig. S12, SI). The strong peaks in the range of 4000 to 4500 cm^−1^ originate from C–H bending as well as C–H stretching vibrations. Bands appearing in the range of 5200 to 5400 cm^−1^ are attributed to C–H overtones. These bands at the range between 5600 and 6200 cm^−1^ arise from the coupling effect between C–H stretch and imidazolium ring vibration along with O–S–O bending vibrations of the [MeSO_4_]^−^ ion. This broadening and shifting of the bands, as seen from Fig. 5, in comparison with pure samples suggests the presence of C_2_–H⋯F and C_2_–H⋯O interactions. This redistribution of the spectra proves that there is cooperative interaction between the anions and cations, and thus the formation of the DSIL and not a mixture can be confirmed. The broadening and shifting of the bands because of the formation of the DSIL from the individual ILs are easier to interpret using the 2D correlation spectroscopic results of their ILs and DSIL. As most of the characteristic peaks were observed between 4000–6500 cm^−1^, the 2D correlation spectra have been plotted in this region only.

The temperature-dependent analysis gives an understanding about the dynamics of the parent ILs and DSILs. The synchronous spectra of the pure [C_4_mim]BF_4_ exhibit a weak autopeak at around 4450 cm^−1^, suggesting that the highly interactive ion pair surroundings are weakly influenced by temperature changes (*cf.* Fig. S13, SI). The off-diagonal cross-peaks between ∼4450 cm^−1^ and ∼5240–5280 cm^−1^ are weak and exhibit mixed signs, suggesting limited coupling between strongly and weakly interacting ionic environments. The asynchronous spectrum further shows only weak cross-peaks between ∼4450 cm^−1^ and ∼5635 cm^−1^, implying minimal sequential or cooperative structural rearrangement. This behavior is consistent with the weakly coordinating nature of the BF_4_^−^ anion, leading to a relatively disordered ionic structure with limited hydrogen bonding and low sensitivity to thermal perturbation. In contrast, pure [C_4_mim][MeSO_4_] exhibits a markedly different behavior (*cf.* Fig. S14, SI). The synchronous spectrum displays a strong autopeak at ∼4430 cm^−1^, indicating that the strongly hydrogen-bonded ion pairs are highly sensitive to temperature changes. The asynchronous spectrum reveals strong and well-defined cross-peaks, particularly between ∼4368 and ∼4444 cm^−1^ and between ∼4420 and ∼5431 cm^−1^. These features indicate a clear sequential evolution of spectral intensities, where changes in strongly hydrogen-bonded environments precede those in weaker interaction regions. This behavior reflects the strong hydrogen-bonding capability of the [MeSO_4_]^−^ anion, which forms a well-organized ionic network that undergoes systematic structural rearrangement with increasing temperature.

The DSIL, [C_4_mim](BF_4_)_0.5_[MeSO_4_]_0.5_, exhibits distinct spectral characteristics that are not a simple superposition of the two parent ILs (*cf.* Fig. S15, SI). The synchronous spectrum shows a strong autopeak at ∼4443 cm^−1^, confirming the presence of strongly interacting ionic environments. However, the appearance of new cross-peaks, particularly between ∼5168 and ∼5274 cm^−1^ and between ∼4443 and ∼5274 cm^−1^, indicates the emergence of additional coupling between different ionic environments. The negative sign of these cross-peaks suggests that the populations of strongly hydrogen-bonded and weakly interacting species vary in opposite directions, pointing to a dynamic equilibrium between these two states. Notably, the coupling observed within the high-wavenumber region (∼5168–5274 cm^−1^) is absent in the pure ILs, indicating the formation of new interaction environments unique to the DSIL system. The asynchronous spectrum of the DSIL provides further compelling evidence for structural reorganization. The strong cross-peak pair at ∼4445 and ∼5226 cm^−1^ reveals a clear sequential relationship between the strongly and weakly interacting species. According to Noda's rule, the positive and negative signs of the cross-peaks indicate that changes in the strongly hydrogen-bonded ion pairs occur prior to those in the weakly interacting ionic environments.^46^ This sequential transformation suggests that temperature initially disrupts strong C_2_–H⋯anion interactions, followed by the formation or enhancement of weaker ionic interactions. Such a well-defined sequence is not observed in the pure systems, highlighting the unique dynamic behavior of the DSIL. The presence of new cross-peaks, altered coupling patterns, and a unique sequential evolution of spectral features collectively indicate that the two anions are not segregated but instead interact cooperatively within a common structural framework. This leads to the formation of new ionic environments with modified hydrogen-bonding characteristics and dynamic behavior that support the successful formation of the DSIL.

**Fig. 6:**
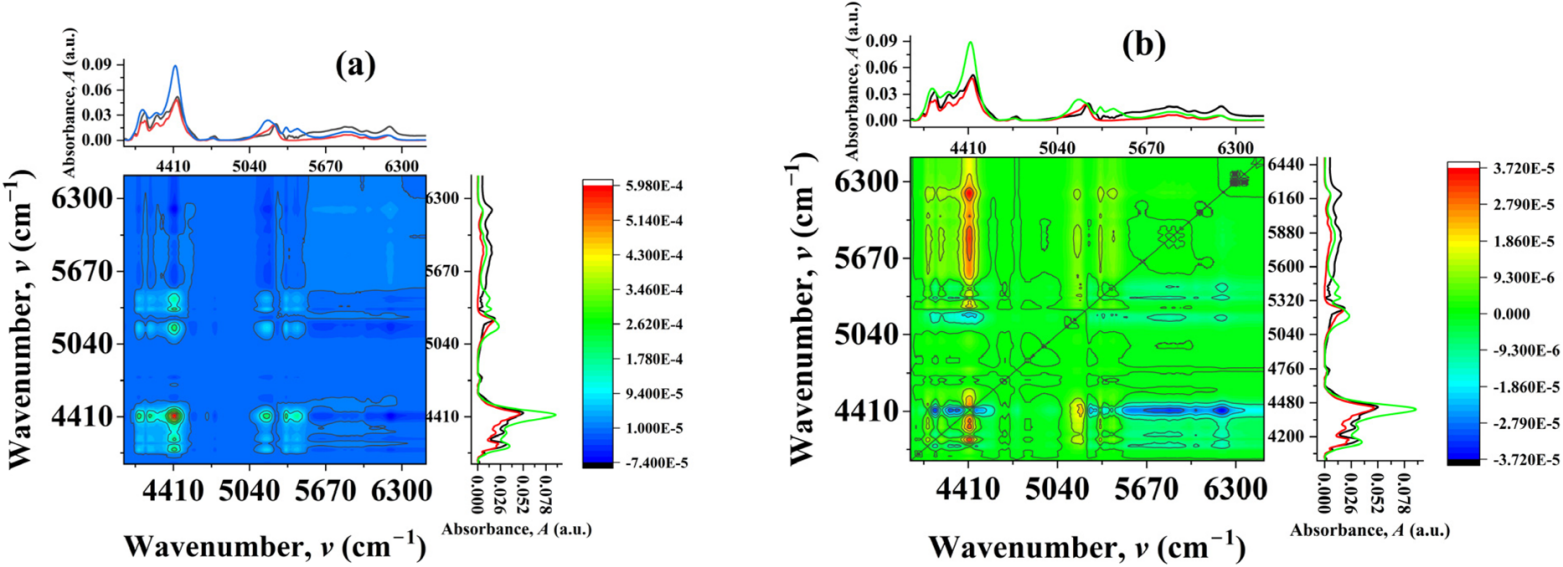
Mole fraction-dependent (a) synchronous and (b) asynchronous 2D correlation spectra of [C_4_mim]BF_4_, [C_4_mim](BF_4_)_0.5_[MeSO_4_]_0.5_, and [C_4_mim][MeSO_4_] where [C_4_mim][MeSO_4_] is progressively introduced into [C_4_mim]BF_4_ (0 → 0.5 → 1.0).

The mole fraction-dependent 2D correlation analysis provides a clear picture of how the ionic structure evolves as [C_4_mim][MeSO_4_] is progressively introduced into [C_4_mim]BF_4_ (0 → 0.5 → 1.0) (Fig. 6). In the NIR region, the dominant diagonal autopeak at ∼4417 cm^−1^ is strong and positive, indicating that this band is the most sensitive to compositional changes. This band is associated with strongly hydrogen-bonded C_2_–H···anion interactions, reflecting tightly associated ion pairs. Additional diagonal features at ∼5183 cm^−1^ and ∼5343 cm^−1^, though weak, correspond to weakly interacting or more “free” ionic environments. The coexistence of these bands across compositions suggests the presence of multiple ionic microenvironments. The synchronous cross-peaks reveal how these environments evolve together with changing composition. Weak positive cross-peaks between ∼4417 cm^−1^ and lower wavenumber features (∼4124–4216 cm^−1^) indicate that closely related strongly interacting environments vary in the same direction, suggesting subtle redistribution within strongly bound structures. Similarly, weak positive correlations between ∼4417 cm^−1^ and ∼5180 cm^−1^ imply that certain strongly and weakly interacting species may initially increase together, reflecting cooperative adjustment during mixing.

However, the dominant feature of the synchronous spectrum is the presence of strong negative cross-peaks between ∼4417 cm^−1^ and higher wavenumber bands (∼5640, 5829, 6035, and 6209 cm^−1^). These negative correlations indicate that as the population of strongly hydrogen-bonded ion pairs changes, the population of weakly interacting or less associated ionic species varies in the opposite direction. This clearly demonstrates a redistribution between strongly coordinated and weakly interacting ionic environments as the mole fraction of [C_4_mim][MeSO_4_] increases. Further insight is obtained from the correlations involving the ∼5190 cm^−1^ band. The strong negative cross-peaks between ∼5190 cm^−1^ and higher wavenumber features (∼5651 and ∼6207 cm^−1^) indicate that even within the weakly interacting region, multiple distinct environments exist and evolve in opposition. This suggests that the addition of [C_4_mim][MeSO_4_] does not simply increase or decrease a single type of weak interaction but redistributes populations among several weakly interacting species. Such behavior is indicative of increasing structural complexity and the formation of new ionic environments as the composition changes. The asynchronous spectrum provides decisive evidence for the sequential nature of these changes. Strong cross-peak pairs such as (4419 cm^−1^, 4293 cm^−1^), (4419 cm^−1^, 4180 cm^−1^), and (4419 cm^−1^, 4475 cm^−1^), with opposite signs across symmetric positions, indicate that spectral intensity changes at ∼4419 cm^−1^ occur earlier than those at neighboring bands in the lower wavenumber region. This implies that strongly hydrogen-bonded ion pairs respond first to compositional perturbation. Similarly, strong asynchronous cross-peaks between ∼4419 cm^−1^ and higher wavenumber bands (∼5829 cm^−1^ and ∼6204 cm^−1^) show that changes in strong interactions precede the development or adjustment of weakly interacting ionic species. This sequential behavior reflects a transformation pathway in which the original ionic network reorganizes before new interaction environments are fully established. The presence of additional weaker asynchronous correlations involving ∼5200 cm^−1^ and ∼5280 cm^−1^ further supports the existence of intermediate or transitional environments that evolve after the primary restructuring of strong ion pairs. The mole fraction-dependent 2D correlation spectra demonstrate that the evolution from pure [C_4_mim]BF_4_ to pure [C_4_mim][MeSO_4_] *via* the equimolar mixture is not a linear or additive process. Instead, the system exhibits strong coupling between different spectral regions, the emergence of new correlation patterns, and a well-defined sequential transformation of ionic environments. This behavior provides compelling molecular-level evidence for the formation of a true DSIL, in which both anions are integrated into a common structural framework rather than existing as independent or segregated species.

Fig. S16 (SI) shows a linear decrease in *ρ* with increasing temperature of binary systems of water and [C_4_mim](BF_4_)_0.5_[MeSO_4_]_0.5_ at 0.1 mole fraction intervals for 0 to 1 mole fraction of the DSIL. There is usually an increase in thermal motion due to an increase in temperature. Eventually, the volume expands and the *ρ* value decreases. In addition to that, at all the temperatures studied here, increasing the water content in the binary mixtures leads to a decline in overall *ρ*. This indicates a significant decrease in molecular-level interactions between DSIL and water, including electrostatic interactions, permanent and induced dipolar forces, dispersion effects, and hydrogen-bonding interactions.^47–49^

### 3.2. Binary mixtures of double salt ionic liquid and water

#### 3.2.1. Volumetric study

The *V*_m_^*E*^ was calculated for binary mixtures comprising strongly hydrogen-bonded water and DSIL, stabilized primarily by coulombic interactions. The experimental values of the *V*_m_^*E*^ are fitted with the following Redlich–Kister type polynomial equation.7
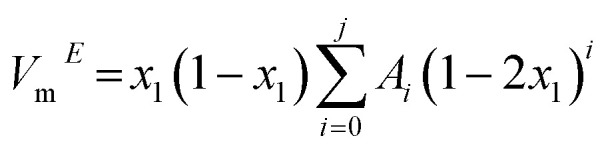
where *A*_*i*_ represents the fitting coefficients determined by the least-squares method with uniform weighting of all data points; *j* denotes the polynomial order; and *x*_1_ corresponds to the mole fraction of DSIL.

**Fig. 7:**
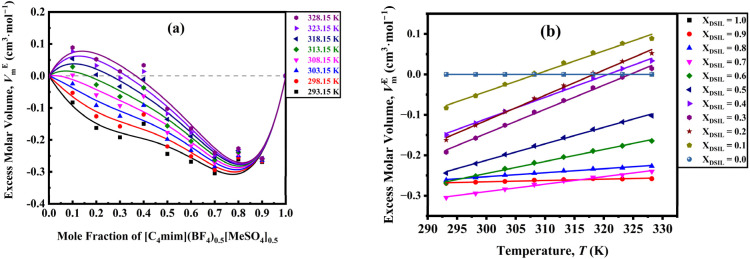
Excess molar volume as a function of (a) mole fraction of [C_4_mim](BF_4_)_0.5_[MeSO_4_]_0.5_ and (b) temperature.

The *V*_m_^*E*^ of [C_4_mim](BF_4_)_0.5_[MeSO_4_]_0.5_-water binary systems with increasing mole fraction of DSIL are shown in Fig. 7. One common feature of all the studied systems is the positive increase of *V*_m_^*E*^ as the temperature is increased. The influence of temperature is more significant at lower DSIL mole fractions than at higher ones.

In an attempt to elucidate the DSIL-water supramolecular interactions from the *V*_m_^*E*^, several competing effects must be considered: (a) breakdown of the intrinsic hydrogen bond structure of water or of the cation and anion of DSIL (electrostatic interactions or van der Waals associations among the constituents, such as alkyl moieties), which gives positive *V*_m_^*E*^ values; (b) ion–polar group interactions between DSIL and water promote close association of the species, resulting in a negative deviation; (c) a packing phenomenon may occur, yielding negative effects when smaller molecules occupy the voids between larger ones, or positive effects when molecular sizes are incompatible.^50^ Only the effect that is predominant over the others for a specific mole fraction range is used to comment on the molecular-level interactions.

For [C_4_mim](BF_4_)_0.5_[MeSO_4_]_0.5_-water binary mixtures, across all compositions, the *V*_m_^*E*^ exhibits mainly negative values. The strong hydrogen-bonding interactions in DSIL-water systems might be responsible for this type of negative deviation. Other contributions may arise from complexation phenomena involving pronounced dipole–dipole interactions between component molecules, driven by the two types of anions in the DSIL. However, in the water-rich region, at around 0.14 mole fraction of DSIL, positive deviations are observed at elevated temperatures for disrupting the hydrogen-bonded water structure and a more pronounced temperature effect. Even in the high DSIL mole fraction region (*x*_1_ ≥ 0.8), DSIL-water associations remain predominant.

Partial molar volume of the components (1 = DSIL and 2 = water) are calculated using,^51,52^8
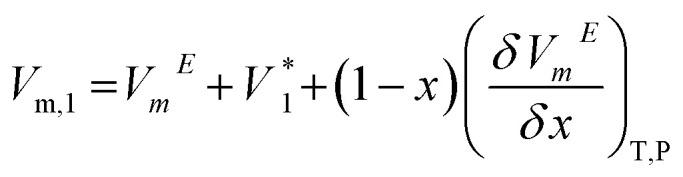
9
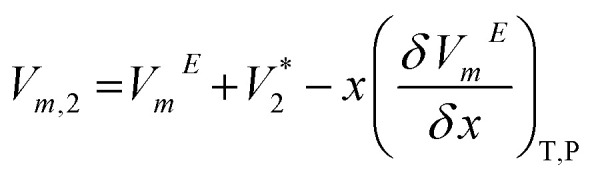
Here, 
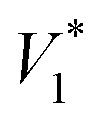
 and 
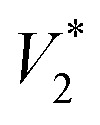
 indicate the molar volumes of components 1 and 2 in their pure states.

Now, the partial molar volume of DSIL at infinite dilution (*V̄*^0^_1_) is calculated using,10
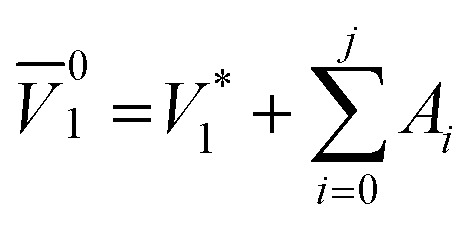
where the coefficients *A*_*i*_ were derived by applying the least-squares method with uniform weighting of data, and *j* is the polynomial degree.

Excess partial molar volume of DSIL (*V*_m,1_^*E*^) is calculated using,11
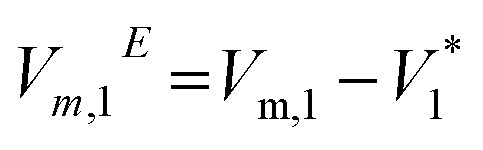


**Fig. 8:**
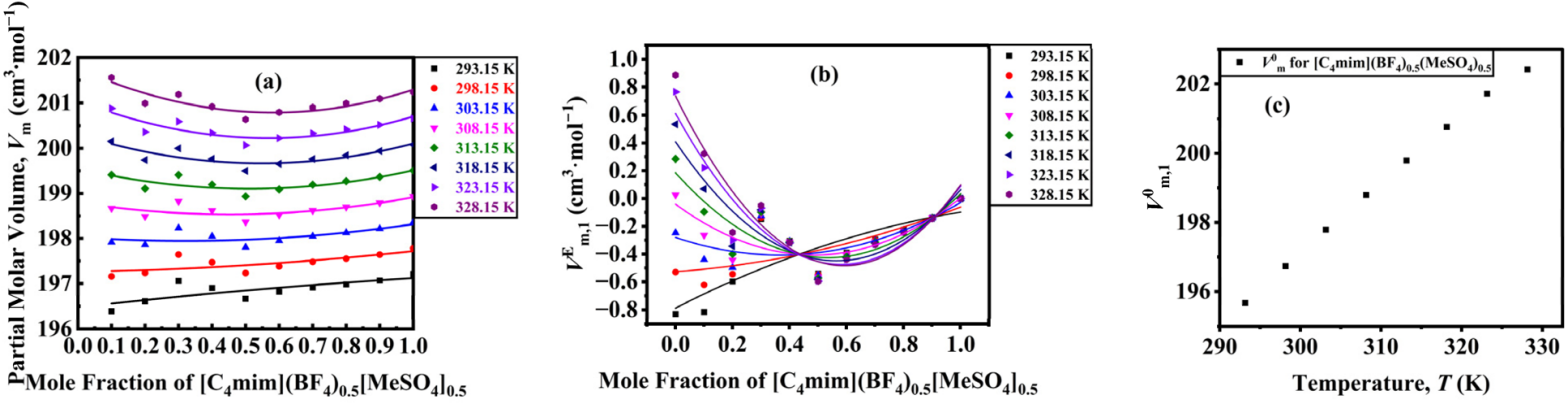
(a) Partial molar volume as a function of mole fraction, (b) excess partial molar volume as a function of mole fraction, and (c) partial molar volume at infinite dilution as a function of temperature for [C_4_mim](BF_4_)_0.5_[MeSO_4_]_0.5_.

The partial molar volume quantifies the volume change resulting from the addition of one mole of a substance to a mixture at constant temperature and pressure.^53^*V*_m_, and *V*_m,1_^*E*^, and *V̄*^0^_1_ as a function of temperature for the binary mixtures, as illustrated in Fig. 8. The order and trend of partial molar volume for all the investigated binary mixtures are in accordance with the results obtained from the *V*_m_^*E*^. Since with increasing mole fraction of DSIL, the more temperature-sensitive water–water interaction weakens, the effect of temperature becomes less significant for the *V*_m,1_^*E*^ in all the studied binary mixtures. In the regime of infinite dilution, the influence of solute–solute interactions can be disregarded, the partial properties provide valuable insights, and *V̄*^0^_1_ reflects solute–solvent interactions without being influenced by composition.^54^ The *V̄*^0^_1_ increases as a function of temperature. This means that the solute–solvent interactions decrease with increasing temperature.

#### 3.2.2. Viscometric study

At all temperatures considered in this study, the *η* of binary systems declines as the water content rises (Fig. S18). These findings align with values previously reported for comparable systems.^55–57^ This has been interpreted in terms of weakening the strong coulombic interaction between like molecules; mixing with the neutral solvent results in enhanced ionic mobility.^58^ The *η* of each mixture was fitted with VFT equation. For comparison, Arrhenius fitting was also performed and yielded good agreement (*R*^2^ ≈ 0.974–0.998) (*cf.* Fig. S17, SI and *cf.* Table S3, SI). However, the VFT model provided consistently superior fits (*R*^2^ ≥ 0.999) (*cf.* Fig. S18, SI and *cf.* Table S4, SI) and better represents the non-Arrhenius behavior associated with the complex, cooperative interactions in IL-water systems;^40–44^ hence, it was adopted for analysis. Temperature exerts a more pronounced influence on *η* than on *ρ*. The *η* in liquids results from molecular cohesion, which explains this behavior. With rising temperature, the molecular vibrations become stronger, the cohesion of molecules becomes weaker, and as a result, *η* goes down.^53^ Another way to look at the reduction of *η* is that the molecules of liquid have a lower resistance to flow at high temperatures. This occurs as molecular kinetic energy rises while intermolecular forces weaken under these conditions. Pure DSIL exhibits a pronounced decline in *η* with temperature, while higher water fractions render the mixtures less responsive to temperature changes. The variation in the *η* of pure water with temperature is minimal compared to that observed for pure DSIL. This phenomenon can be interpreted in terms of the intensity of interactions between molecules in the liquid. Forces such as ionic interactions, ion–dipole attractions, hydrogen bonding, and dipole–dipole interactions, hold molecules together in a liquid. Stronger interaction causes higher sensitivity towards temperature. According to the microscopic structural model, in the clusters of ions, cations and anions are connected through hydrogen bonds to form a quasi-three-dimensional network, where the cations form successive layers, and the anions occupy the interlayer spaces. Cluster formation enhances the structural rigidity of the DSIL, which is absent in water molecules. The *E* values for DSIL and their binary systems with water were calculated at 298 K and illustrated in Fig. S19 (SI).

For the binary mixtures of water and [C_4_mim](BF_4_)_0.5_[MeSO_4_]_0.5_, the *E* values rise upon increasing the DSIL content and become almost constant after 0.8 mole fraction. It shows a maximum value of 15.57 kJ mol^−1^.

**Fig. 9:**
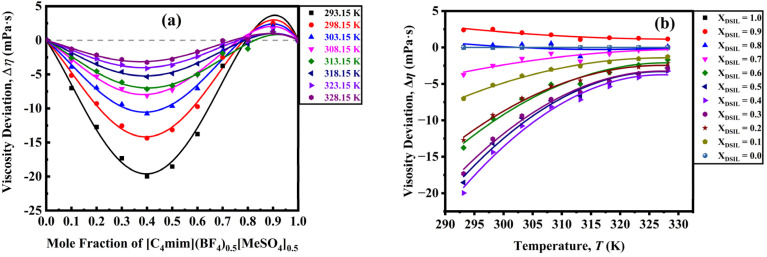
Viscosity deviation as a function of (a) mole fraction and (b) temperature for [C_4_mim](BF_4_)_0.5_[MeSO_4_]_0.5_.

The Δ*η* for the DSIL-water binary mixtures was determined and subsequently correlated using a Redlich–Kister type polynomial.12
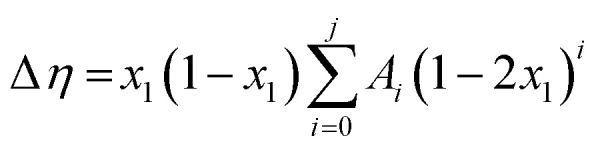
where *A*_*i*_ represents the polynomial coefficients determined by the least-squares method with equal weighting of all data points, and *j* denotes the polynomial degree. The Δ*η* provides a qualitative measure of the interaction strength within the binary mixtures. Regarding its interpretation, the following factors can be considered:^59^ (a) the reduction in *η* may result from variations in the spatial form and volume of the components, along with the disruption of dipolar associations in the pure substances, resulting in a negative Δ*η* value, and (b) specific interactions between different components, including hydrogen bonding and charge-transfer complex formation, may lead to higher *η* in the mixtures compared to the pure substances that produce positive values for Δ*η*. Generally speaking, systems with dispersion and dipole–dipole interactions have negative Δ*η* values, but charge-transfer interactions and hydrogen bonds cause complex species to develop between dissimilar molecules, which results in positive Δ*η* values.^60^ [C_4_mim](BF_4_)_0.5_[MeSO_4_]_0.5_-water binary mixtures show positive deviation for *x*_DSIL_ ≥ 0.8 and negative deviation in the remaining mole fraction ranges (Fig. 9). It is possible to interpret the negative Δ*η* of the studied mixtures as a sign that molecular attraction forces outweigh repulsive forces. Eventually, the attraction forces are predominant at intermediate mole fractions. The positive values at the DSIL-rich region indicate that the hydrogen-bonding interactions between DSIL and water predominate in this region. The effect of temperature also becomes less significant in this region. The values of experimental *ρ*, *η*, calculated *V*_m_^*E*^, partial molar volume (*V*_m,1_), and Δ*η* for [C_4_mim](BF_4_)_0.5_[MeSO_4_]_0.5_-water binary mixtures are listed in Table S5.

The flow of a liquid is determined by how easily molecules can move into available voids and how readily the liquid forms such voids,^61^ is controlled by the Δ*G*. Fig. S20(a) (SI) exhibits that the positive Δ*G* values for the binary mixtures decrease with higher temperatures and reduced DSIL mole fractions for all of the binary mixtures. This indicates the process is endergonic, and increasing temperature and lower DSIL content facilitate easier movement of DSIL molecules throughout the mixture. The reduction in Δ*G* with increasing temperature is likely caused by a reduction in DSIL-water interactions. This result supports the one obtained for the change in *V̄*^0^_1_ with increasing temperature. The Δ*S* was calculated using the derivative of free energy change, −d(Δ*G*)/d*T* from Fig. S20(a) (SI). The Δ*S* at all temperatures, despite some discontinuities (*cf.* Fig. S20(b), SI), decreases with the elevation of temperature and reduction of mole fraction of DSIL. Therefore, the elevation of temperature and reduction of mole fraction make the system organized. The positive Δ*H* values reveal that dissolving DSIL in water is an endothermic process. Fig. S20(c) (SI) shows that the Δ*H* decreases with higher temperatures and lower DSIL contents. In essence, as the DSIL mole fraction rises, Δ*H* increases, reducing molecular mobility and leading to a higher activation energy. So, upon elevating the temperature and reducing the DSIL mole fraction, the mobility is increasing. The observation aligns with hole theory, which suggests that solvent flow depends on the creation of transient holes.^62^

### 3.3. Variation of the size of aggregates with varying mole fractions of DSIL in DSIL-water systems

DLS analysis was conducted to characterize the size of clusters formed in DSIL-water binary systems. The internal arrangement of the liquid system can be directly inferred from particle size analysis^.^ Throughout the entire composition range, the DSIL-water binary systems exhibited three distinct aggregate sizes (*cf.* Fig. S21, SI). The first type of aggregates formed at around 0.6–1.0 nm and did not shift much with an elevated content of water. The second type of cluster shifted position with higher water content and ranged from 1.3 to 68.1 nm. The third type of cluster was present at around 4150.0–5560.0 nm, which did not shift position to a significant extent. The appearance of this third species may suggest interactions between the DSIL cation and anions. In regions with high DSIL content, water molecules are confined within the polar network formed by DSIL cation and anions. With increasing mole fraction of water, large water clusters are formed, which percolate the whole system. The water-rich region shows a moderate degree of association before the DSILs are fully dissolved. Water molecules may be located at the interstice formed by the cation and anions of DSIL.

### 3.4. Variation of the size of aggregates with varying temperatures for DSIL-water binary systems

In [C_4_mim](BF_4_)_0.5_[MeSO_4_]_0.5_-water binary mixtures, three different types of clusters are observed. With increasing temperature, clusters with larger hydrodynamic diameters decrease in intensity. It was also observed that the polydispersity of the cluster sizes of ILs increases with increasing temperature, although the sizes become smaller. This implies that the large aggregates break down to form smaller aggregates of varying sizes. The range of sizes exhibited by the aggregates ^in^ [C_4_mim](BF_4_)_0.5_[MeSO_4_]_0.5_-water binary systems are shown in Fig. S22 (SI) (*X*_DSIL_ = 0.9, 0.8, 0.7, 0.6, 0.5, 0.4, 0.3, and 0.1).

### 3.5. Temperature-dependent near-infrared spectroscopic measurements of DSIL-water binary mixtures

NIR spectroscopic measurements were performed on pure water, pure [C_4_mim](BF_4_)_0.5_[MeSO_4_]_0.5_, and their binary systems with water, with the mole fraction of *X*_DSIL_ = 0.9 to 0.1 from 20 to 70 °C at 5 °C intervals. Fig. S23 and S24 (SI) show the NIR spectra of pure water and pure [C_4_mim](BF_4_)_0.5_[MeSO_4_]_0.5_. Water possesses three characteristic vibrational frequencies corresponding to symmetric stretching (*v*_1_), bending (*v*_2_), and asymmetric stretching (*v*_3_). For a free water molecule, these vibrations appear at 3651.7 cm^−1^, 1595.0 cm^−1^, and 3755.8 cm^−1^, respectively. While the bending mode shows minimal dependence on hydrogen bonding, the stretching vibrations are strongly impacted by the intensity of nearby hydrogen-bonding interactions. The NIR spectrum of pure water shows two broad bands, one for the combination of the O–H *v*_2_ and *v*_3_ and the other for the combination of the O–H *v*_1_ and *v*_3_. Various other combinations and overtones exist in this region, which overlap with the aforementioned bands. The temperature-dependent NIR spectra of liquid water show a general increase in absorbance and blue shifting of both bands with increasing temperature. Two isosbestic points were observed at 5188 cm^−1^ and 6939 cm^−1^. NIR spectra showed bands around 4000–4800 cm^−1^ and around 5500–6400 cm^−1^. Some bands around 6450–7650 cm^−1^ were also observed. Signals near 4168 cm^−1^ arose from the combined C–H stretching and bending vibrations. The band at 5882 cm^−1^ arises from the imidazolium C_2_–H band. The combination of aromatic C_4,5_−H shows a band around 6203 cm^−1^. The peak around 5000–5400 cm^−1^ shows the most prominent and clear change. Hence, the spectral region between 5000 and 5400 cm^−1^ was selected for detailed analysis to elucidate the supramolecular interactions in the DSIL-water mixtures. Section S1 (SI) represents the 2D correlation spectroscopy of pure water.

### 3.6. 2D Correlation analysis of [C_4_mim](BF_4_)_0.5_[MeSO_4_]_0.5_-water binary mixtures

The influence of temperature on hydrogen bonding of different types of water species from 0.9 to 0.1 mole fraction of DSIL in [C_4_mim](BF_4_)_0.5_[MeSO_4_]_0.5_-water binary mixtures were investigated using a 2D correlation spectroscopic approach. For the sake of comparison, only the 2D plots of pure [C_4_mim](BF_4_)_0.5_[MeSO_4_]_0.5_ and [C_4_mim](BF_4_)_0.5_[MeSO_4_]_0.5_-water binary mixtures with *X*_DSIL_ = 0.9, 0.5 and 0.1 are shown. Synchronous and asynchronous correlation diagrams for pure [C_4_mim](BF_4_)_0.5_[MeSO_4_]_0.5_ are shown in Fig. S26(a) and (b) (SI).

**Fig. 10:**
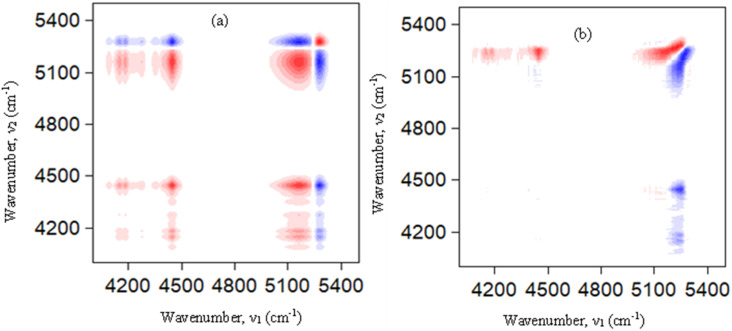
(a) Synchronous and (b) asynchronous plot for 0.5 [C_4_mim](BF_4_)_0.5_[MeSO_4_]_0.5_-water binary mixture.

The synchronous correlation map exhibits several auto-peaks. There was the presence of multiple cross-peaks with both positive and negative values as well. The asynchronous diagram also showed two peaks showing maxima at 4455 cm^−1^ and 5226 cm^−1^, and minima at 5249 cm^−1^ and 5243 cm^−1^. This suggests that the spectral variations at 4455 cm^−1^ and 5226 cm^−1^ precede those observed at 5249 cm^−1^ and 5243 cm^−1^. Synchronous and asynchronous correlation diagram for [C_4_mim](BF_4_)_0.5_[MeSO_4_]_0.5_-water binary mixtures with *X*_DSIL_ = 0.9 are presented in Fig. S27(a) and (b) (SI). In this case, the synchronous map shows four auto-peaks at 4163 cm^−1^, 4444 cm^−1^, 5179 cm^−1^, and 5300 cm^−1^. Other than these, there were several cross-peaks with both positive and negative correlations. This indicates the presence of different clusters in this system. The asynchronous map showed numerous off-diagonal peaks which are similar to [C_4_mim](BF_4_)_0.5_[MeSO_4_]_0.5_-water binary mixtures with *X*_DSIL_ = 0.9. Synchronous and asynchronous correlation diagram for [C_4_mim](BF_4_)_0.5_[MeSO_4_]_0.5_-water binary mixtures with *X*_DSIL_ = 0.5 are shown in Fig. 10(a) and (b). Positive auto-peaks were observed at 5191 cm^−1^ and 5296 cm^−1^ in the synchronous map. This suggests that the spectral signals at these positions are interrelated and change synchronously. Two negative cross-peaks at 5179 cm^−1^ and 5284 cm^−1^ are shown as well. This predicts that the changes are occurring in opposite directions.

**Fig. 11:**
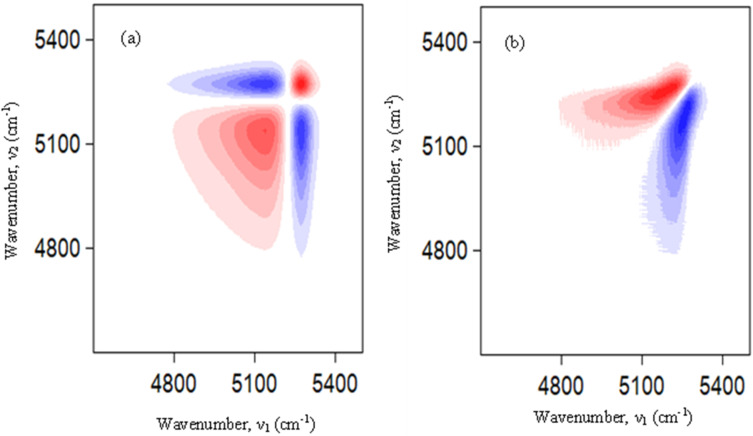
(a) Synchronous and (b) asynchronous plot for 0.1 [C_4_mim](BF_4_)_0.5_[MeSO_4_]_0.5_- water binary mixture.

The asynchronous correlation spectrum exhibits two features, with maxima at 5177 cm^−1^ and minima at 5257 cm^−1^, which indicates that changes occur at 5177 cm^−1^ before 5257 cm^−1^. Synchronous and asynchronous correlation diagram for [C_4_mim](BF_4_)_0.5_[MeSO_4_]_0.5_-water binary mixtures with *X*_DSIL_ = 0.1 are shown in Fig. 11(a) and (b)^.^ Two auto-peaks at 5138 cm^−1^ and 5282 cm^−1^ are in the synchronous map. The intensity of weakly hydrogen-bonded species is greater than that of strongly hydrogen-bonded species. Between the auto-peaks, two negative cross-peaks are noted. This implies that the correlated changes happen in reverse directions. The asynchronous map showed spectral features with minimum and maximum values at 5185 and 5267 cm^−1^. The sign of these peaks indicates that spectral variations are occurring asynchronously at 5185 cm^−1^ before 5267 cm^−1^.

### 3.7. Supramolecular interactions between pure ionic liquids, double salt ionic liquid, and double salt ionic liquid-water binary systems

A key distinction between DSILs and conventional IL mixtures lies in their physicochemical response. DSILs frequently exhibit pronounced non-ideal behavior, reflected in excess properties such as *V*_m_^*E*^ and Δ*η*, arising from complex ion–ion, ion–dipole, and hydrogen-bonding interactions. In addition, the coexistence of chemically distinct ions within a shared ionic network can lead to enhanced structural heterogeneity and dynamic microstructuring. As a result, transport properties such as viscosity can deviate significantly from those of the parent ILs, often showing reduced viscosity and improved mobility due to more efficient packing and cooperative interactions. These features contrast with more ideal or near-ideal behavior that may be observed in simple IL mixtures lacking strong specific interactions. The supramolecular interactions in pure ILs and the DSIL arise from a subtle interplay of electrostatic, hydrogen-bonding, dipolar, and dispersive forces that dictate their microscopic organization and macroscopic fluidity (*cf.* Fig. S28, SI). In pure [C_4_mim]BF_4_ and [C_4_mim][MeSO_4_], strong electrostatic attractions between the imidazolium cation [C_4_mim]^+^ and their respective counterions dominate the liquid structure. The BF^−^_4_ anion, being small, symmetrical, and weakly coordinating, promotes efficient cation–anion packing and compact ionic domains stabilized mainly by coulombic and van der Waals interactions. In contrast, [MeSO_4_]^−^ is larger, more asymmetric, and capable of forming directional hydrogen bonds with the acidic C_2_–H site of the imidazolium ring, leading to stronger and more localized ion pairing, greater structural heterogeneity, and higher *η*. Upon equimolar mixing of these two ILs to form the DSIL, [C_4_mim](BF_4_)_0.5_[MeSO_4_]_0.5_, the coexistence of chemically and geometrically distinct anions introduces competing interactions within the ionic network. The weakly coordinating BF_4_^−^ and the strongly hydrogen-bonding [MeSO_4_]^−^ create a heterogeneous electrostatic environment that disrupts the uniform packing of ions observed in the parent ILs. This results in the partial breakdown of long-range cation–anion correlations, formation of additional free volume, and enhancement of local structural disorder. The cooperative interplay between the two anions induces a balance between electrostatic and dispersive interactions, giving rise to a more dynamically flexible liquid matrix with reduced *η* and lower activation barriers for flow. With increasing temperature, the supramolecular organization undergoes further modification. Thermal agitation weakens the strong coulombic and hydrogen-bonding interactions that dominate at lower temperatures, leading to increased molecular mobility and disruption of transient ion pairs. In pure ILs, this results in the gradual breakdown of well-organized ion domains and a linear decrease in *η*, *ρ*, and activation enthalpy. In the DSIL, however, the pre-existing structural heterogeneity allows for greater adaptability; as temperature rises, the mixed-anion environment accommodates thermal fluctuations more efficiently, reducing structural cooperativity without fully destroying the local microstructure.

**Fig. 12:**
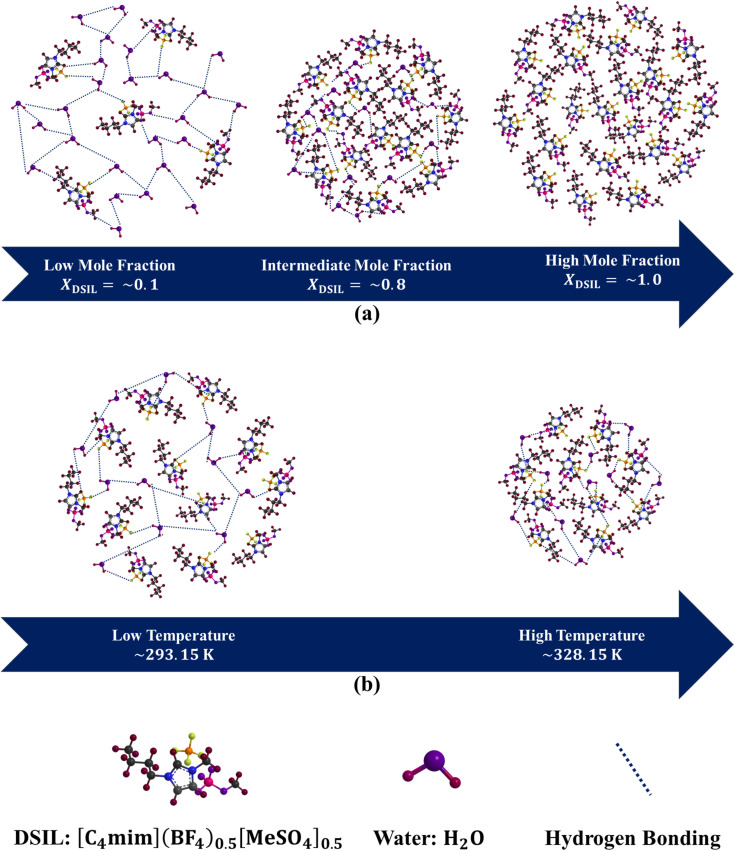
Molecular-level interactions between DSIL and water at (a) various mole fractions of DSIL and (b) various temperatures in the binary mixtures of DSIL and water.

Significant molecular-level interactions between DSIL and water have been found in DSIL-water binary mixtures (Fig. 12). These interactions include hydrogen bonds, dispersion forces, dipole-induced dipole interactions, and coulombic forces. These interactions depend on temperature as well as the mole fraction of DSIL. Compared to the high DSIL mole fraction region, the low DSIL mole fraction zone is more affected by temperature. A relatively small amount of DSIL causes the 3D hydrogen-bonded network to be disrupted. As the mole fraction of DSIL increases, the degree of hydrogen bonding rises until it reaches a certain (around 0.8) mole fraction of DSIL, beyond which the hydrogen bonding with water becomes weaker. Likewise, the strong coulombic interactions among the ions in DSIL that form rigid cluster structures start diminishing upon adding water. The DSIL-water interactions decrease with increasing temperature. The influence of temperature decreases as the mole fraction of DSIL increases because the more temperature-sensitive water–water interaction gets less. Among the three types of clusters that are formed in the binary mixtures, the smallest hydrodynamic populations (0.6–1.0 nm) are tentatively attributed to water-rich domains, whereas the larger aggregates (4150.0–5560.0 nm) likely correspond to DSIL-enriched ionic assemblies.

The intermediate-sized clusters (1.3–68.1 nm) having both DSIL and water in a significant amount are prominent up to 0.8 mole fraction of DSIL, and the number of these clusters starts diminishing above this mole fraction. It should be noted that DLS provides hydrodynamic diameters of scattering species and does not directly yield their chemical composition. Therefore, the assignment of specific size populations to particular components have been made cautiously and within the context of the system under investigation. In the present binary system, comprising water and the DSIL, the two components differ markedly in molecular size, structural complexity, and interaction strength. Water forms relatively small, transient hydrogen-bonded assemblies, whereas the DSIL consists of bulky ions capable of generating extended, strongly interacting ionic networks. Such interpretations are well supported by previous DLS studies on aqueous systems containing molecular solutes.^28–30^

The results underpin a molecular basis for DSIL with specific physicochemical characteristics for desired applications. Future developments of next-generation functional fluids for energy storage systems, adjustable electrolytes, smart solvents for biomass valorization, green chemical processes, and sophisticated heat transfer and lubrication technologies are made possible by this research. Future research can improve the knowledge of these adaptable solvent systems by expanding the investigation to include more DSIL and cosolvent combinations and examining how they behave under confinement or when functional components (such as nanoparticles or polymers) are present. Finally, these systems are positioned as prospective candidates for applications in responsive materials, sustainable technologies, and molecular engineering platforms due to their capacity to accurately tune bulk characteristics and intermolecular interactions through DSIL-solvent composition and heat control.

## 4. Conclusion

The transformation of pure ionic liquids into a double salt ionic liquid alters the balance of supramolecular interactions, converting a rigid, strongly correlated ionic network into a more flexible and dynamically disordered structure. The coexistence of weakly coordinating (BF_4_^−^) and strongly hydrogen-bonding ([MeSO_4_]^−^) anions disrupts uniform cation–anion packing, introduces free volume, and reduces long-range electrostatic correlations, resulting in lower *η* and activation barriers. Temperature further modulates these interactions by weakening coulombic and hydrogen-bonding forces, enhancing molecular mobility, and inducing microstructural reorganization near 308–313 K. Overall, the DSIL exhibits a thermally responsive, less cohesive supramolecular framework with improved fluidity and tunable dynamic behavior compared to its parent ILs.

Supramolecular interactions between DSIL and water evolve non-linearly with composition and temperature. A chaotropic nature is observed in both water-rich (around 0.1 mole fraction of DSIL) and DSIL-rich (around 0.9 mole fraction of DSIL) regions in the binary mixtures of DSIL and water. However, a kosmotropic nature is observed in the intermediate region (0.5–0.8 mole fraction of DSIL) of the mixture. The weakening of self-association among water occurs upon the addition of DSIL and leads to the formation of small clusters (0.6–1.0 nm). The weakening of the self-association of DSIL occurs upon the addition of water in a similar fashion, leading to the formation of large clusters (4150.0–5560.0 nm). The ion-polar group attraction forces bring the species close together, and the liquid gets structured, and intermediate-sized clusters (1.3–68.1 nm) are formed. The size of intermediate-sized clusters varies with the mole fraction of DSIL among the three cluster types and it goes through a minimum with increasing amounts of DSIL.

Water exists in multiple hydrogen-bonding states, transitioning through a quasi-two-state model. Also, the hydrogen bonding is disrupted with increasing temperature and DSIL concentration, with the spectral changes indicating a shift from strongly to weakly hydrogen-bonded species. The low DSIL mole fraction region experiences a greater temperature influence than the high DSIL mole fraction region, and the hydrogen-bonded water structure is disrupted at high temperatures. This work provides critical insight into the molecular organization and interaction mechanisms within DSIL-water systems. These findings have potential implications for the rational design of IL-based solvents and functional media where control over solvation, aggregation, and hydrogen bonding is essential.

## Author contributions

K. M Golam Rahman: conducting experiments, data curation, validation, writing – original draft. Mohammad Hossain: data curation, validation, writing – original draft, review, and editing. Md. Abu Bin Hasan Susan: conceptualization, visualization, resources, writing – review and editing, supervision, project administration, funding acquisition.

## Conflicts of interest

There are no conflicts to declare.

## Supplementary Material

RA-016-D6RA00801A-s001

## Data Availability

Data supporting this article have been included as part of the supplementary information (SI). Further data may be available upon request. Supplementary information: variation of density (*ρ*), dynamic viscosity (*η*), free energy (Δ*G*), entropy (Δ*S*), and enthalpy (Δ*H*) changes of activation for viscous flow as a function of temperature for pure ILs, [C_4_mim]BF_4_ and [C_4_mim][MeSO_4_] and DSIL, [C_4_mim](BF_4_)_0.5_[MeSO_4_]_0.5_; and as a function of temperature and mole fraction for the binary mixtures of water and DSIL. Calculated *V*_m_^*E*^, viscosity deviation (Δ*η*), energy barrier (*E*) for DSIL and its component ILs. Experimental *ρ*, *η*, calculated *V*_m_^*E*^, partial molar volume (*V*_m,1_), and Δ*η* for DSIL-water binary mixtures at different temperatures. Size distribution of aggregates formed in DSIL-water binary mixture at various temperatures and mole fractions. NIR spectra, synchronous, and asynchronous spectra of pure water and pure DSIL. See DOI: https://doi.org/10.1039/d6ra00801a.
