# Supplementary material for: Molecular-level interactions governed by temperature and composition in double salt ionic liquid-water systems
Source: RSC Adv. 2026 Jun 4;16(33):30741–58. doi: 10.1039/d6ra00801a (PMC13237657; doi:10.1039/d6ra00801a)
Supplement: RA-016-D6RA00801A-s001 [file RA-016-D6RA00801A-s001.pdf]

Supplementary Information (SI) for RSC Advances.
This journal is © The Royal Society of Chemistry 2026

Molecular-Level Interactions Governed by Temperature and Composition in Double Salt Ionic Liquid–Water Systems

K M Golam Rahman,[a] Mohammad Hossain,[ab] and Md. Abu Bin Hasan Susan*[ac]

[a]*Department of Chemistry, University of Dhaka, Dhaka 1000, Bangladesh;*

[b]*Department of Chemistry, Bangladesh University of Engineering and Technology (BUET), Dhaka 1000, Bangladesh;*

[c]*Dhaka University Nanotechnology Center (DUNC), University of Dhaka, Dhaka 1000, Bangladesh*

* Corresponding author. susan@du.ac.bd

Electronic Supplementary Information

Table of Contents

No. of Fig.	Caption of Figure	Page
**Fig. S1**	Densities of $[C_4mim]BF_4$, $[C_4mim][MeSO_4]$, and $[C_4mim](BF_4)_{0.5}[MeSO_4]_{0.5}$ as a function of temperature.	S4
**Fig. S2**	Arrhenius plot of ln(η) versus 1/T for $[C_4mim]BF_4$, $[C_4mim][MeSO_4]$, and $[C_4mim](BF_4)_{0.5}[MeSO_4]_{0.5}$ showing linear behavior over the studied temperature range and corresponding regression fits.	S4
**Table S1**	Arrhenius fitting parameters (intercept and slope) and corresponding coefficients of determination (R^2) obtained from linear regression of ln(η) versus 1/T for $[C_4mim]BF_4$, $[C_4mim][MeSO_4]$, and $[C_4mim](BF_4)_{0.5}[MeSO_4]_{0.5}$	S5
**Fig. S3**	Viscosities of $[C_4mim]BF_4$, $[C_4mim][MeSO_4]$, and $[C_4mim](BF_4)_{0.5}[MeSO_4]_{0.5}$ as a function of temperature with Vogel–Fulcher–Tammann (VFT) fitting.	S5
**Table S2**	Vogel–Fulcher–Tammann (VFT) fitting parameters (A, B, and x_0) and corresponding coefficients of determination (R^2) obtained from fitting the temperature dependence of viscosity (η) for $[C_4mim]BF_4$, $[C_4mim][MeSO_4]$, and	S6

$[C_4mim](BF_4)_{0.5}[MeSO_4]_{0.5}$

Fig. S4 Energy barrier values of $[C_4mim]BF_4$, $[C_4mim][MeSO_4]$, and $[C_4mim](BF_4)_{0.5}[MeSO_4]_{0.5}$ as a function of temperature. S6

Fig. S5 Changes in entropy of activation for the viscous flow of $[C_4mim]BF_4$, $[C_4mim][MeSO_4]$, and $[C_4mim](BF_4)_{0.5}[MeSO_4]_{0.5}$ as a function of temperature. S7

Fig. S6 Changes in enthalpy of activation for the viscous flow of $[C_4mim]BF_4$, $[C_4mim][MeSO_4]$, and $[C_4mim](BF_4)_{0.5}[MeSO_4]_{0.5}$ as a function of temperature. S7

Fig. S7 Size of aggregates formed in pure $[C_4mim]BF_4$ at various temperatures. S8

Fig. S8 Size of aggregates formed in pure $[C_4mim][MeSO_4]$ at various temperatures. S8

Fig. S9 Size of aggregates formed in $[C_4mim](BF_4)_{0.5}[MeSO_4]_{0.5}$ at various temperatures. S9

Fig. S10 NIR spectra of pure $[C_4mim]BF_4$ at various temperatures. S9

Fig. S11 NIR spectra of pure $[C_4mim][MeSO_4]$ at various temperatures. S10

Fig. S12 NIR spectra of pure $[C_4mim](BF_4)_{0.5}[MeSO_4]_{0.5}$ at various temperatures. S10

Fig. S13 Temperature-dependent (a) synchronous and (b) asynchronous 2D correlation spectra of pure $[C_4mim]BF_4$. S11

Fig. S14 Temperature-dependent (a) synchronous and (b) asynchronous 2D correlation spectra of pure $[C_4mim][MeSO_4]$. S12

Fig. S15 Temperature-dependent (a) synchronous and (b) asynchronous 2D correlation spectra of pure $[C_4mim](BF_4)_{0.5}[MeSO_4]_{0.5}$. S13

Fig. S16 Density as a function of temperature and mole fraction for $[C_4mim](BF_4)_{0.5}[MeSO_4]_{0.5}$ and water binary mixtures. S14

**Fig. S17**	Arrhenius plot of ln(η) versus 1/T for DSIL, $[C_4mim](BF_4)_{0.5}[MeSO_4]_{0.5}$-water binary mixtures showing linear behavior over the studied temperature range and corresponding regression fits.	S15
**Table S3**	Arrhenius fitting parameters (intercept and slope) and corresponding coefficients of determination (R^2) obtained from linear regression of ln(η) versus 1/T for $[C_4mim](BF_4)_{0.5}[MeSO_4]_{0.5}$-water binary mixtures	S15
**Fig. S18**	Viscosity as a function of **(a)** temperature with VFT fitting and **(b)** mole fraction for $[C_4mim](BF_4)_{0.5}[MeSO_4]_{0.5}$-water mixtures.	S16
**Table S4**	Vogel–Fulcher–Tammann (VFT) fitting parameters (A, B, and x_0) and corresponding coefficients of determination (R^2) obtained from fitting the temperature dependence of viscosity (η) for $[C_4mim](BF_4)_{0.5}[MeSO_4]_{0.5}$-water binary mixtures	S17
**Fig. S19**	Calculated energy barrier for DSIL and its component ILs.	S18
**Table S5**	Experimental densities (ρ), dynamic viscosities (η), calculated V_m^E, partial molar volume ($V_{m,1}$), and viscosity deviation ($\Delta\eta$) for $[C_4mim](BF_4)_{0.5}[MeSO_4]_{0.5}$-water binary mixtures at different temperatures	S19
**Fig. S20**	(a) Free energy, (b) entropy, and (c) enthalpy changes of activation for viscous flow as a function of temperature and mole fraction for the binary mixtures of water and $[C_4mim](BF_4)_{0.5}[MeSO_4]_{0.5}$.	S24
**Fig. S21**	Size distribution of aggregates formed in $[C_4mim](BF_4)_{0.5}[MeSO_4]_{0.5}$-water binary mixture from X_{DSIL} = 0.9 to 0.1 at 25 °C.	S25
**Fig. S22**	Size distribution of aggregates formed in $[C_4mim](BF_4)_{0.5}[MeSO_4]_{0.5}$-water binary mixture for X_{DSIL} = 0.9, 0.8, 0.7, 0.6, 0.5, 0.4, 0.3, and 0.1 at several temperatures from 20 to 40 °C.	S26-S27
**Fig. S23**	NIR spectra of pure water from 20 to 70 °C.	S28
**Fig. S24**	NIR spectra of pure $[C_4mim](BF_4)_{0.5}[MeSO_4]_{0.5}$ from 20 to 70 °C.	S28
**Section S1**	2D correlation Analysis of Pure Water	S29
**Fig. S25**	(a) Synchronous and (b) asynchronous plot for pure water.	S29

**Fig. S26**	(a) Synchronous and (b) asynchronous plot for pure $[C_4mim](BF_4)_{0.5}[MeSO_4]_{0.5}$.	S30
**Fig. S27**	(a) Synchronous and (b) asynchronous plot for 0.9 $[C_4mim](BF_4)_{0.5}[MeSO_4]_{0.5}$-water binary mixture.	S31
**Fig. S28**	Molecular-level interactions when (a) pure ILs are combined to form DSIL and (b) temperature increases gradually for DSIL.	S32

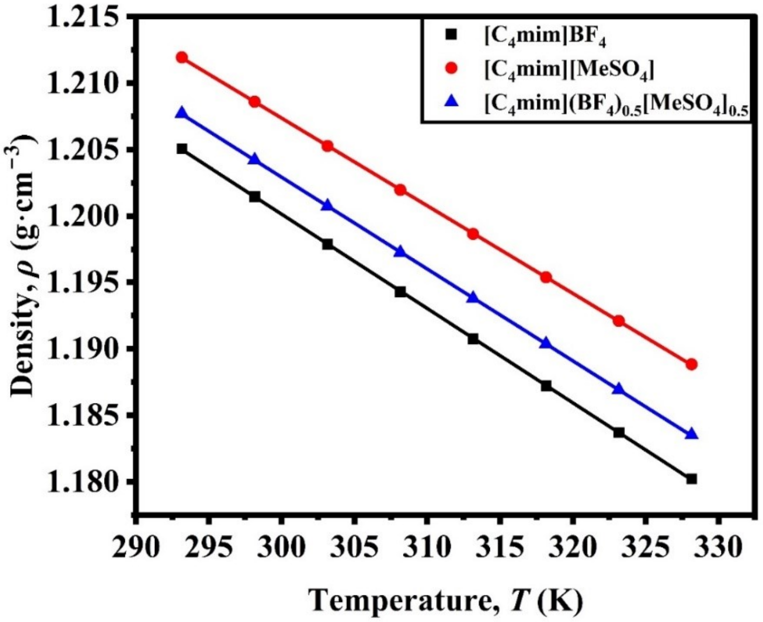

Fig. S1 Densities of $[C_4mim]BF_4$, $[C_4mim][MeSO_4]$, and $[C_4mim](BF_4)_{0.5}[MeSO_4]_{0.5}$ as a function of temperature.

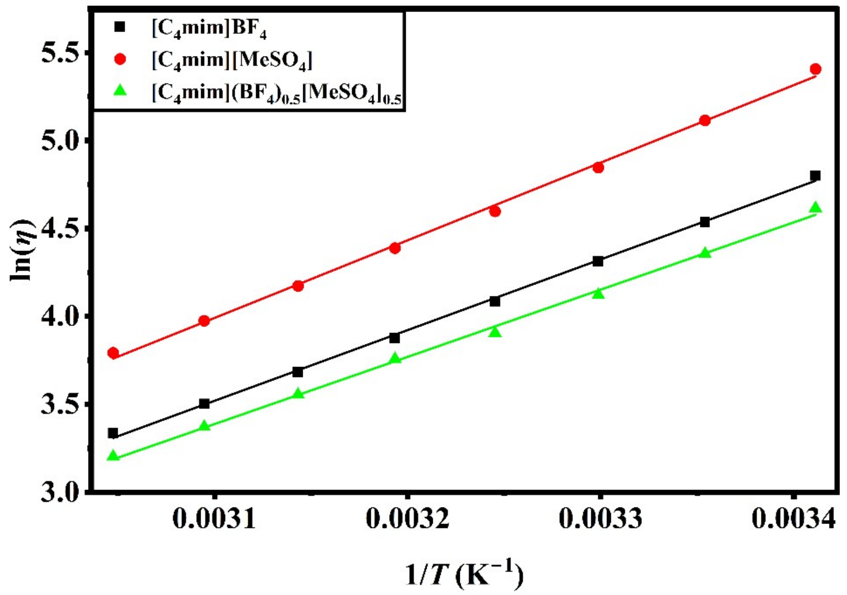

Fig. S2 Arrhenius plot of ln(η) versus $1/T$ for $[C_4mim]BF_4$, $[C_4mim][MeSO_4]$, and $[C_4mim](BF_4)_{0.5}[MeSO_4]_{0.5}$ showing linear behavior over the studied temperature range and corresponding regression fits.

Table S1. Arrhenius fitting parameters (intercept and slope) and corresponding coefficients of determination (R^2) obtained from linear regression of ln(η) versus $1/T$ for $[C_4mim]BF_4$, $[C_4mim][MeSO_4]$, and $[C_4mim](BF_4)_{0.5}[MeSO_4]_{0.5}$

Ionic Liquids and Double Salt Ionic Liquid	Intercept	Slope	R^2
$[C_4mim]BF_4$	-8.937	4018.598	0.998
$[C_4mim][MeSO_4]$	-9.682	4411.155	0.997
$[C_4mim](BF_4)_{0.5}[MeSO_4]_{0.5}$	-8.457	3821.252	0.997

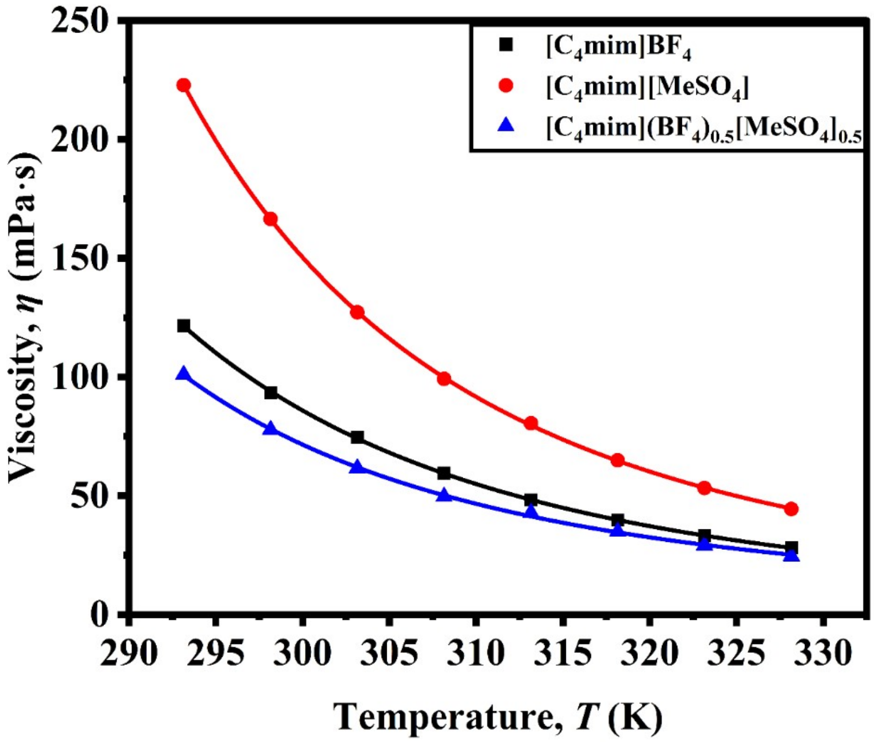

Fig. S3 Viscosities of $[C_4mim]BF_4$, $[C_4mim][MeSO_4]$, and $[C_4mim](BF_4)_{0.5}[MeSO_4]_{0.5}$ as a function of temperature with Vogel–Fulcher–Tammann (VFT) fitting.

Table S2. Vogel–Fulcher–Tammann (VFT) fitting parameters (*A*, *B*, and x_0) and corresponding coefficients of determination (R^2) obtained from fitting the temperature dependence of viscosity (η) for $[C_4mim]BF_4$, $[C_4mim][MeSO_4]$, and $[C_4mim](BF_4)_{0.5}[MeSO_4]_{0.5}$

Ionic Liquids and Double Salt Ionic Liquid	A	B	x_0	R^2
$[C_4mim]BF_4$	-0.887	382.067	164.563	0.999
$[C_4mim][MeSO_4]$	-0.442	292.877	188.194	0.999
$[C_4mim](BF_4)_{0.5}[MeSO_4]_{0.5}$	-0.317	232.191	193.095	0.999

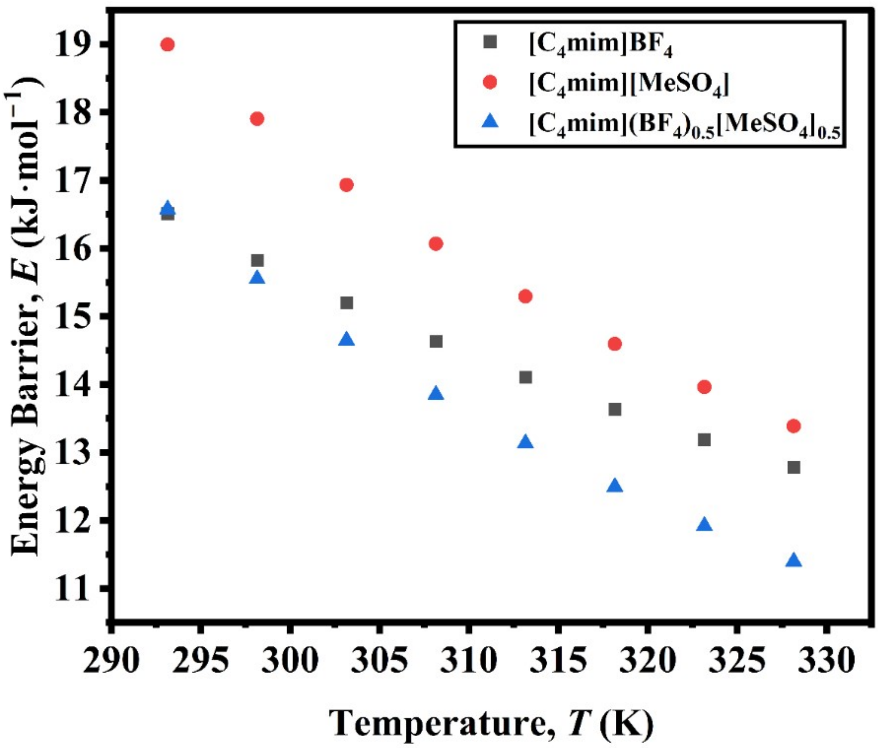

Fig. S4 Energy barrier values of $[C_4mim]BF_4$, $[C_4mim][MeSO_4]$, and $[C_4mim](BF_4)_{0.5}[MeSO_4]_{0.5}$ as a function of temperature.

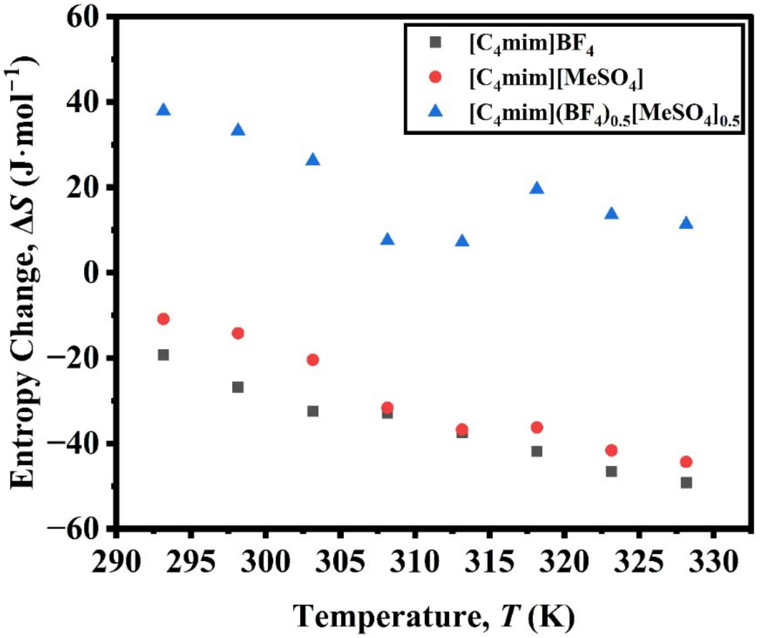

Fig. S5 Changes in entropy of activation for the viscous flow of $[C_4mim]BF_4$, $[C_4mim][MeSO_4]$, and $[C_4mim](BF_4)_{0.5}[MeSO_4]_{0.5}$ as a function of temperature.

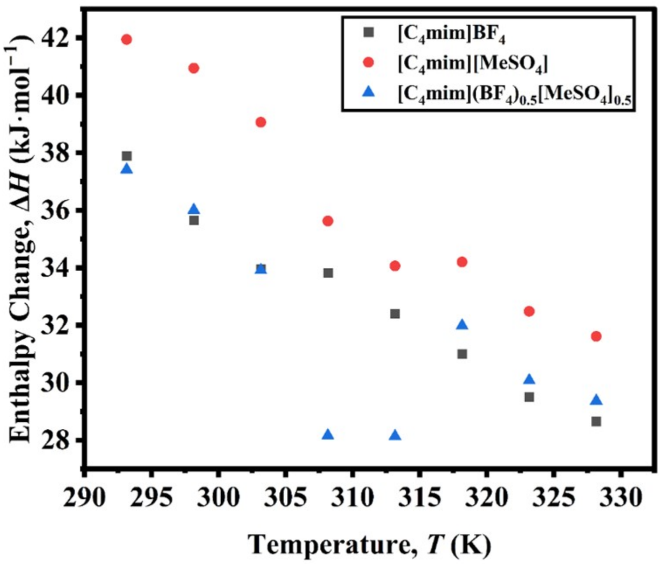

Fig. S6 Changes in enthalpy of activation for the viscous flow of $[C_4mim]BF_4$, $[C_4mim][MeSO_4]$, and $[C_4mim](BF_4)_{0.5}[MeSO_4]_{0.5}$ as a function of temperature.

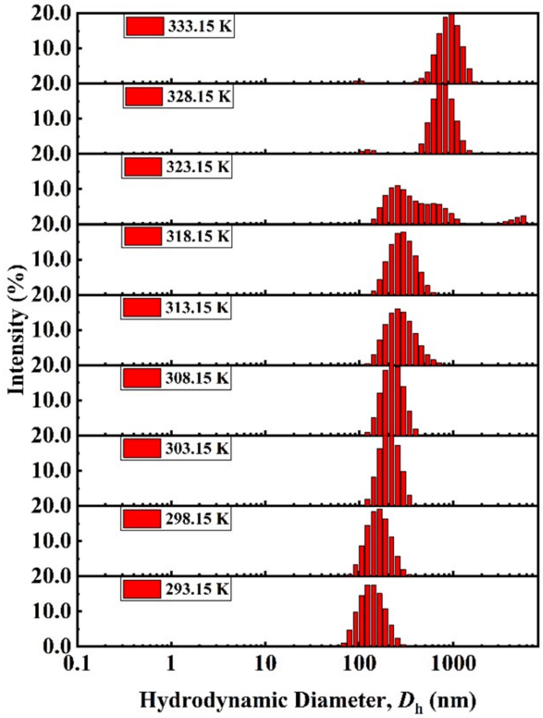

Fig. S7 Size of aggregates formed in pure $[C_4mim]BF_4$ at various temperatures.

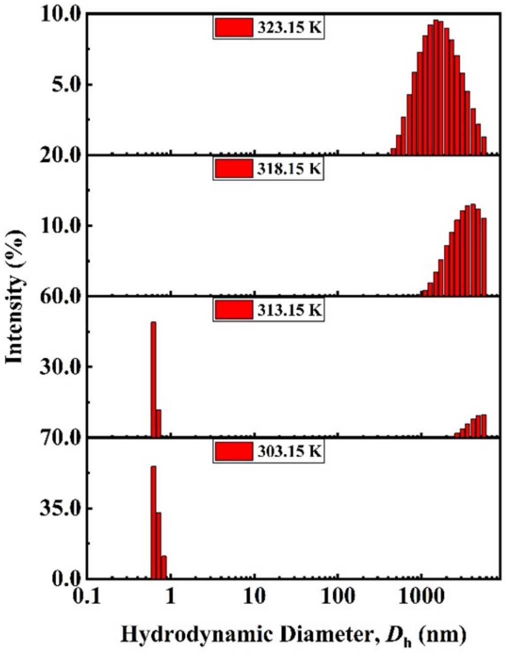

Fig. S8 Size of aggregates formed in pure $[C_4mim][MeSO_4]$ at various temperatures.

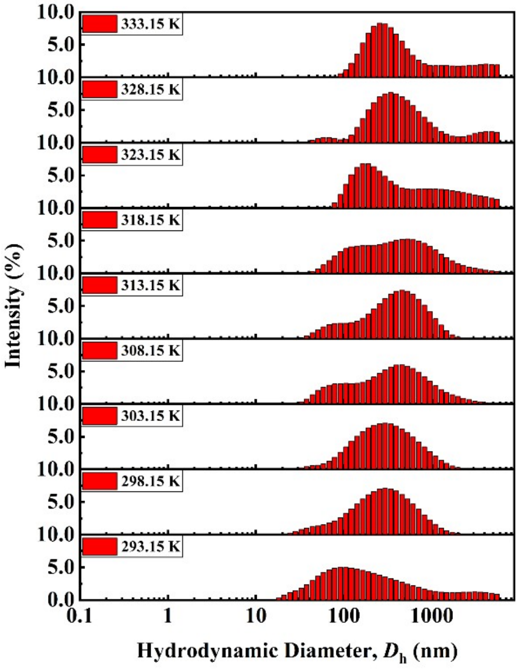

Fig. S9 Size of aggregates formed in $[C_4mim](BF_4)_{0.5}[MeSO_4]_{0.5}$ at various temperatures.

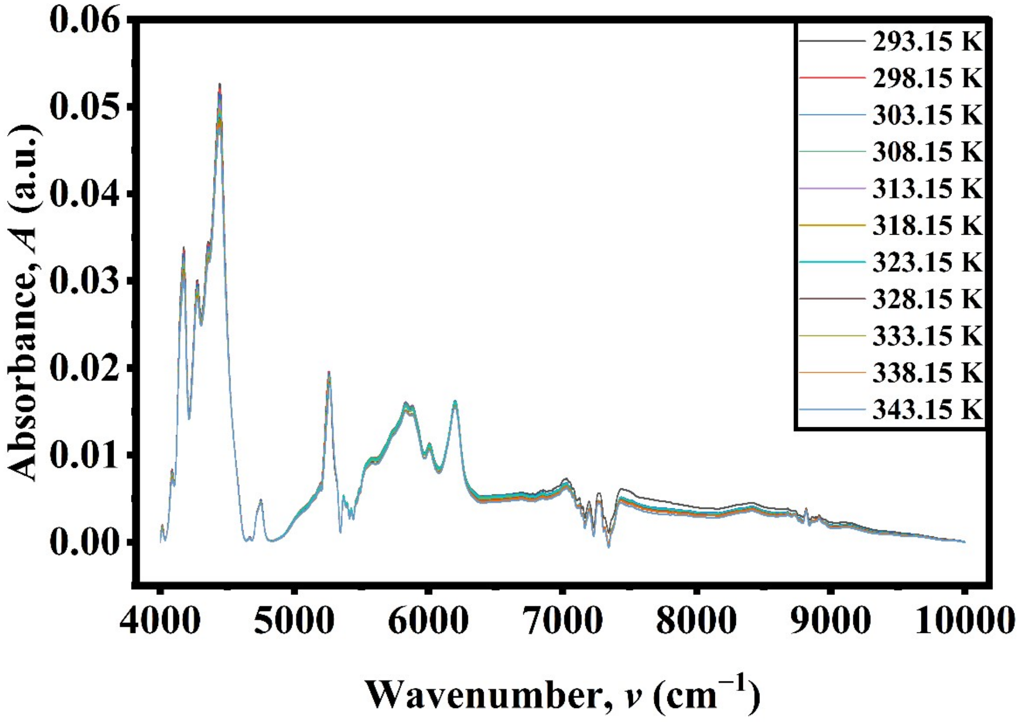

Fig. S10 NIR spectra of pure $[C_4mim]BF_4$ at various temperatures.

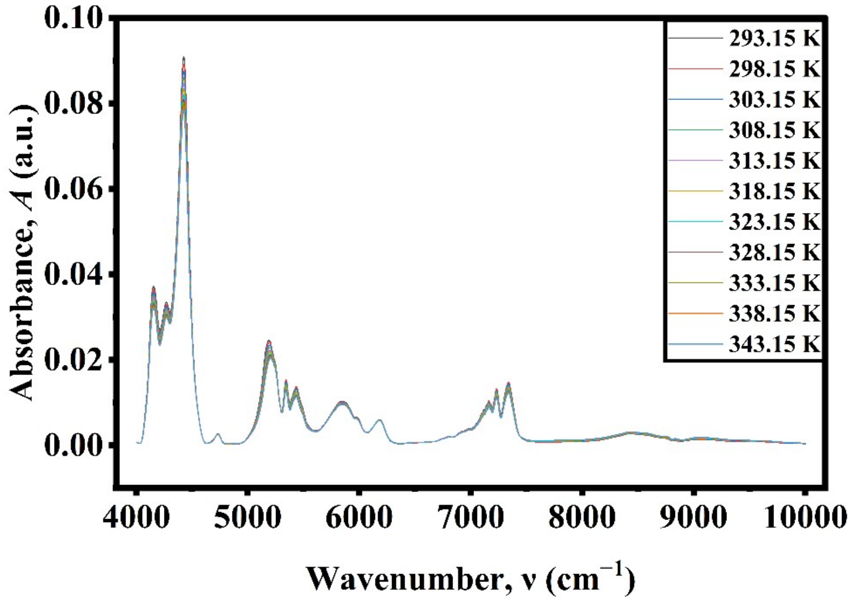

Fig. S11 NIR spectra of pure $[C_4mim][MeSO_4]$ at various temperatures.

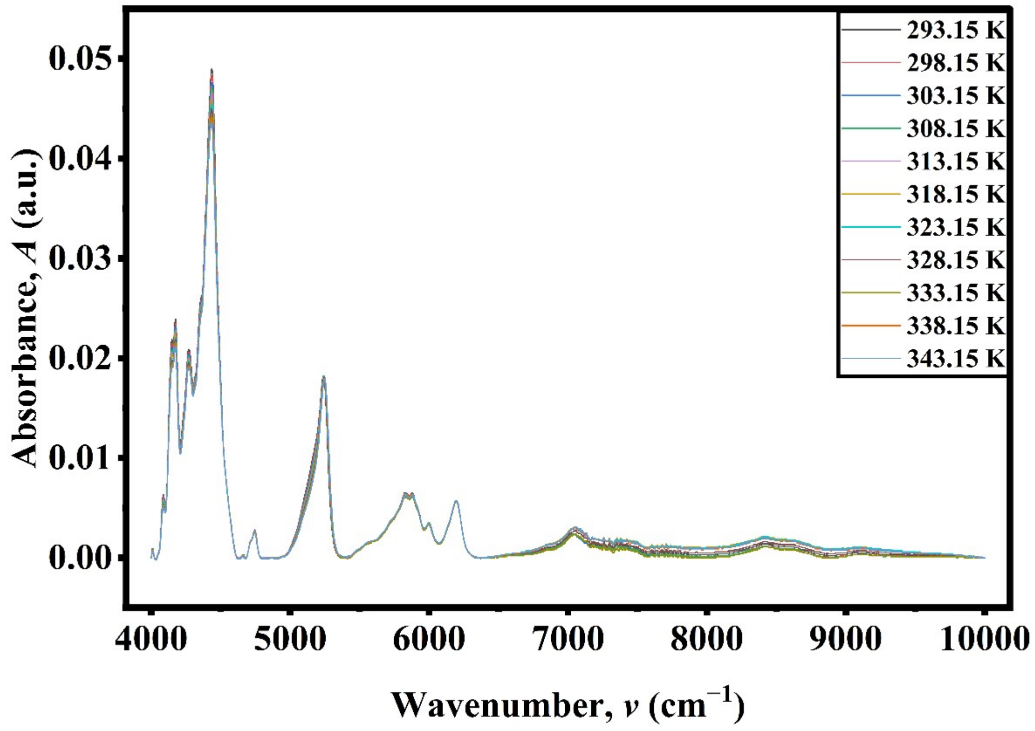

Fig. S12 NIR spectra of pure $[C_4mim](BF_4)_{0.5}[MeSO_4]_{0.5}$ at various temperatures.

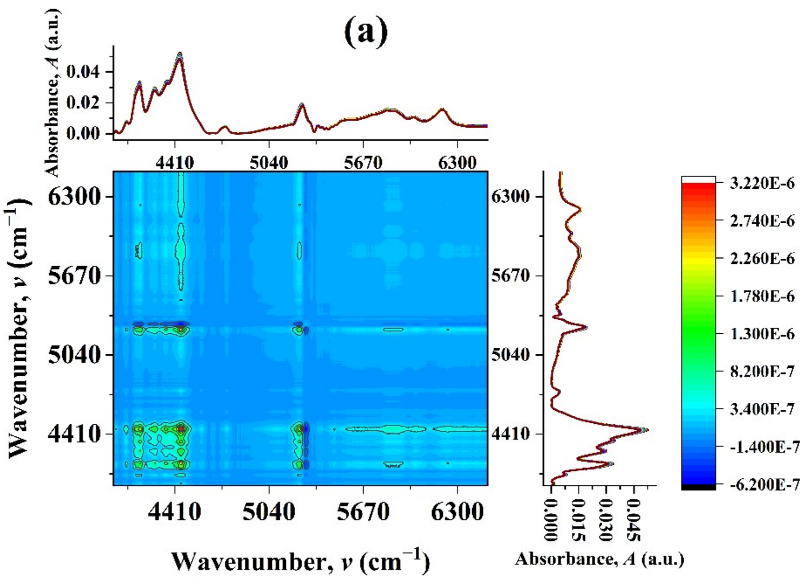

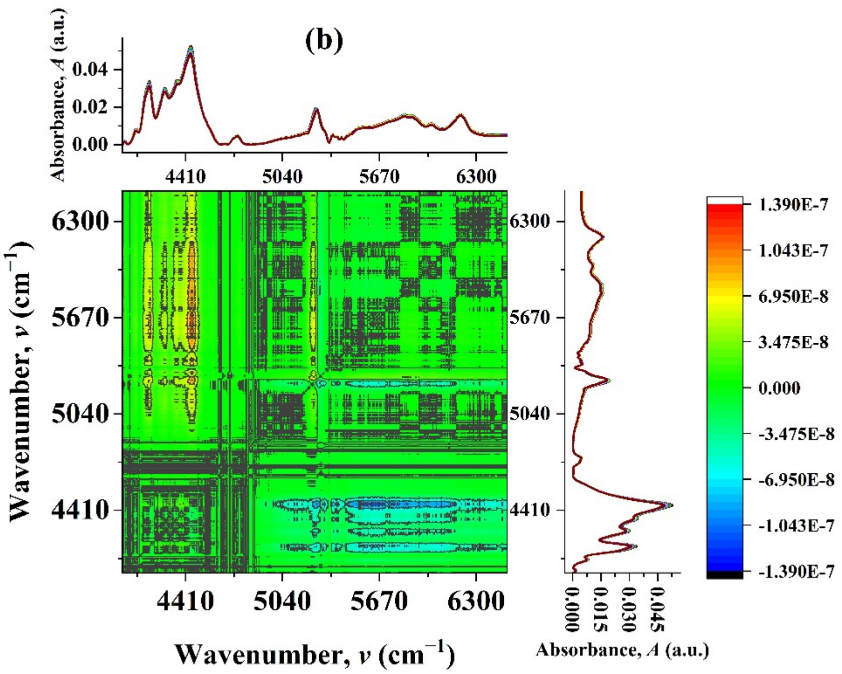

Fig. S13 Temperature-dependent (a) synchronous and (b) asynchronous 2D correlation spectra of pure $[C_4mim]BF_4$.

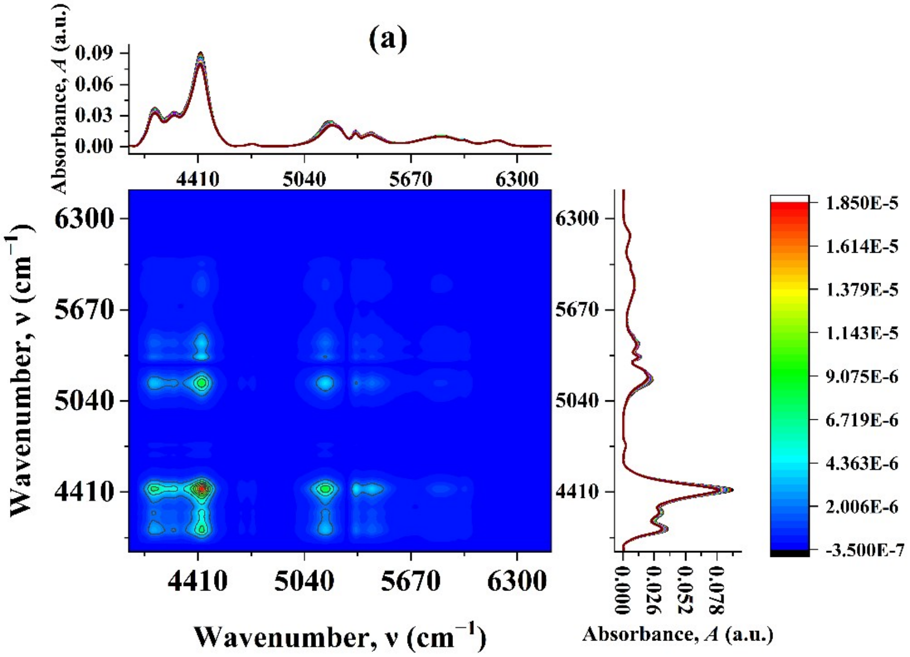

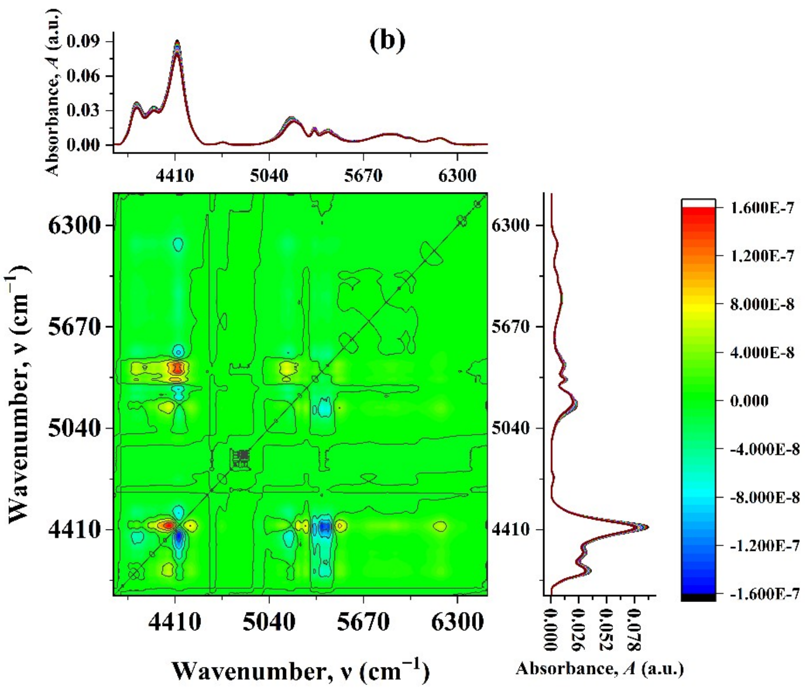

Fig. S14 Temperature-dependent (a) synchronous and (b) asynchronous 2D correlation spectra of pure $[C_4mim][MeSO_4]$.

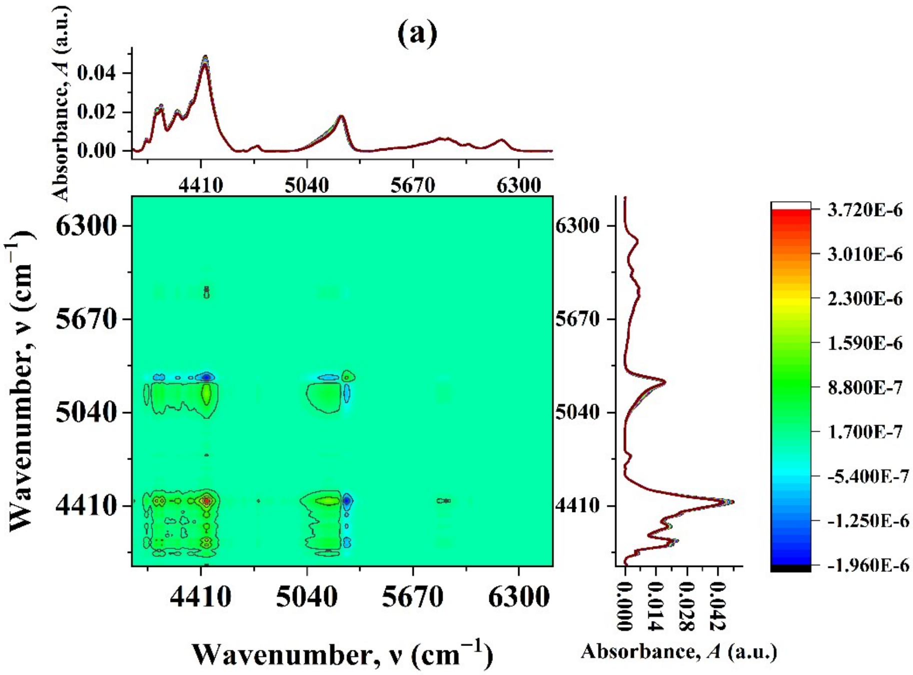

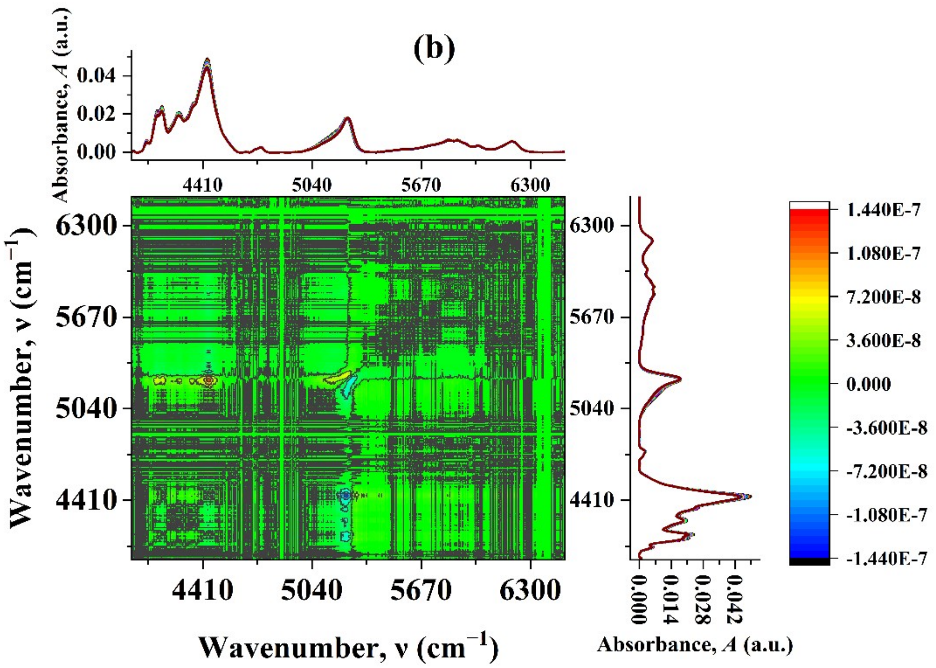

Fig. S15 Temperature-dependent (a) synchronous and (b) asynchronous 2D correlation spectra of pure $[C_4mim](BF_4)_{0.5}[MeSO_4]_{0.5}$.

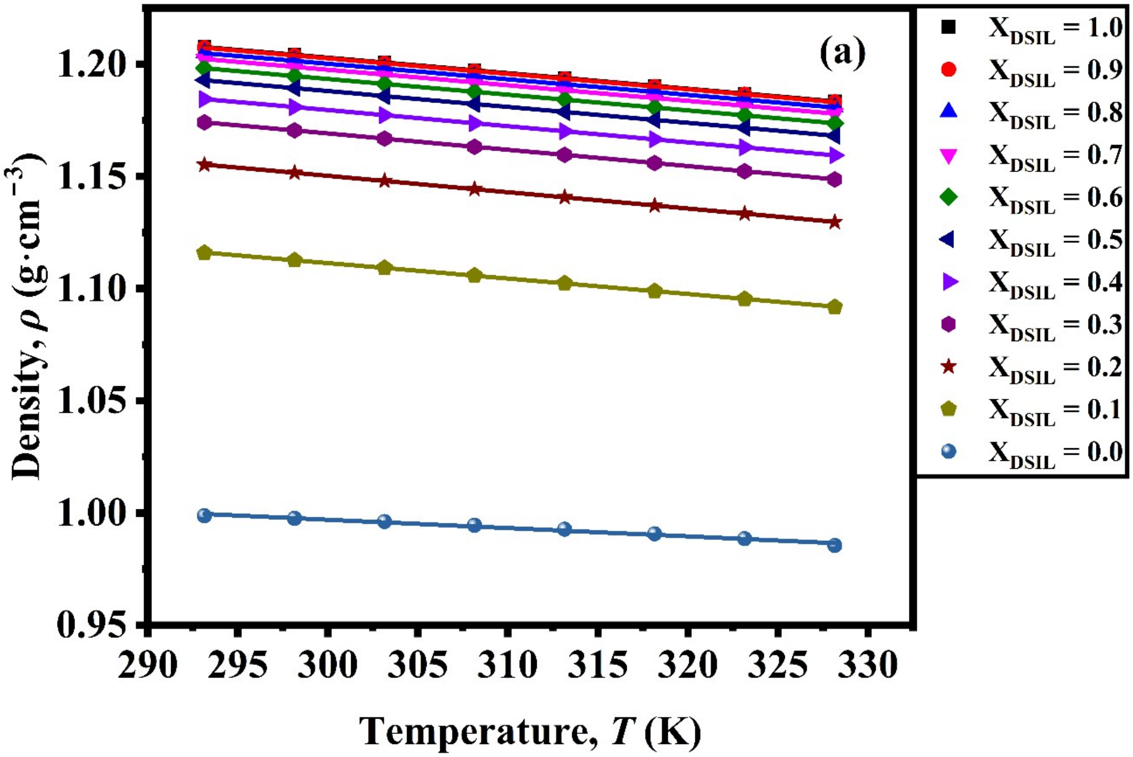

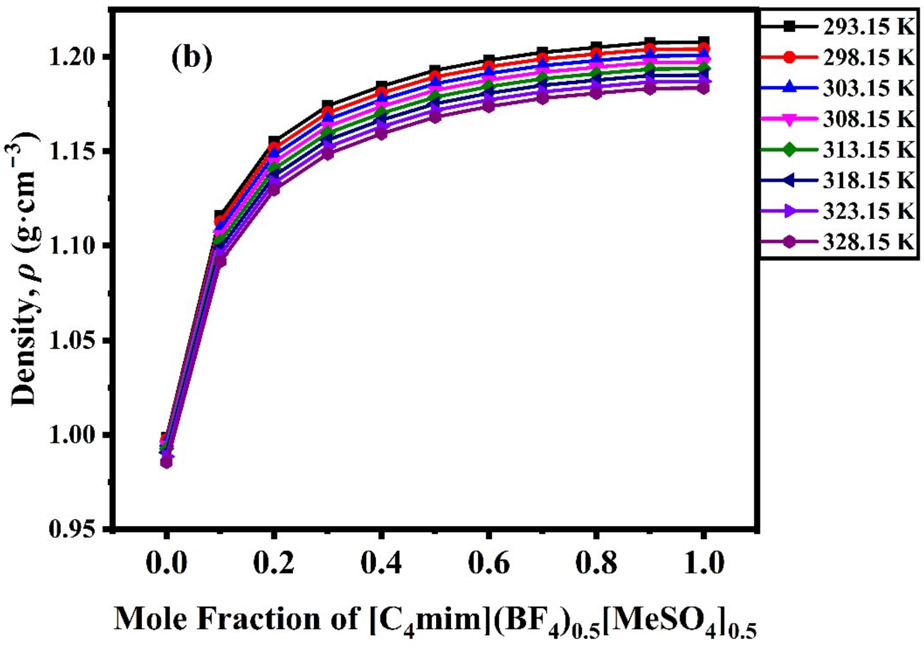

Fig. S16 Density as a function of (a) temperature and (b) mole fraction for $[C_4mim](BF_4)_{0.5}[MeSO_4]_{0.5}$ and water binary mixtures.

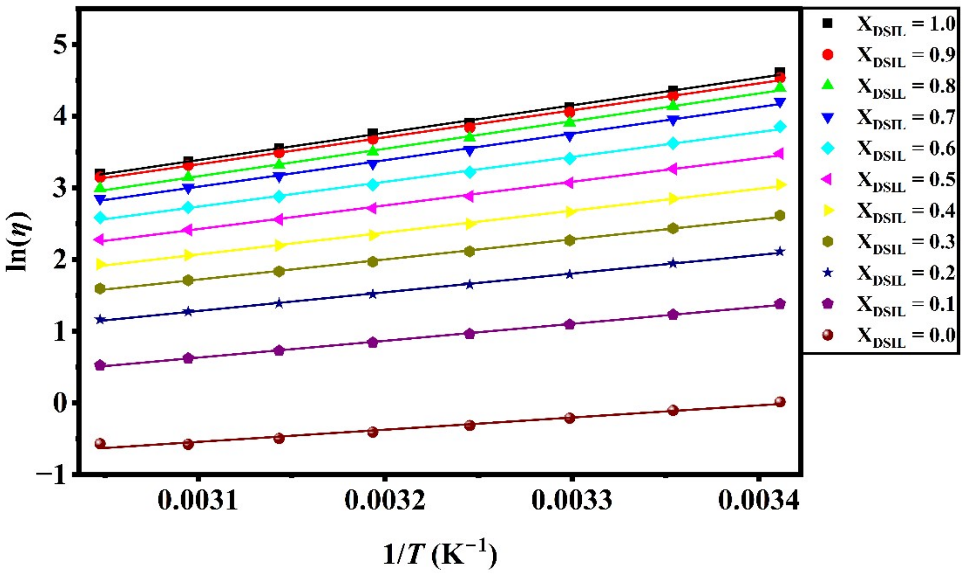

Fig. S17 Arrhenius plot of ln(η) versus $1/T$ for DSIL, $[C_4mim](BF_4)_{0.5}[MeSO_4]_{0.5}$-water binary mixtures showing linear behavior over the studied temperature range and corresponding regression fits.

Table S3. Arrhenius fitting parameters (intercept and slope) and corresponding coefficients of determination (R^2) obtained from linear regression of ln(η) versus $1/T$ for $[C_4mim](BF_4)_{0.5}[MeSO_4]_{0.5}$-water binary mixtures

X_{DSIL}	Intercept	Slope	R^2
1.0	-8.457	3821.252	0.997
0.9	-8.335	3762.926	0.997
0.8	-8.766	3848.134	0.997
0.7	-8.467	3704.680	0.997
0.6	-8.008	3467.086	0.996
0.5	-7.795	3297.423	0.997
0.4	-7.380	3049.440	0.997
0.3	-6.951	2798.375	0.998
0.2	-6.788	2604.343	0.998
0.1	-6.670	2356.289	0.998
0.0	-5.807	1698.961	0.974

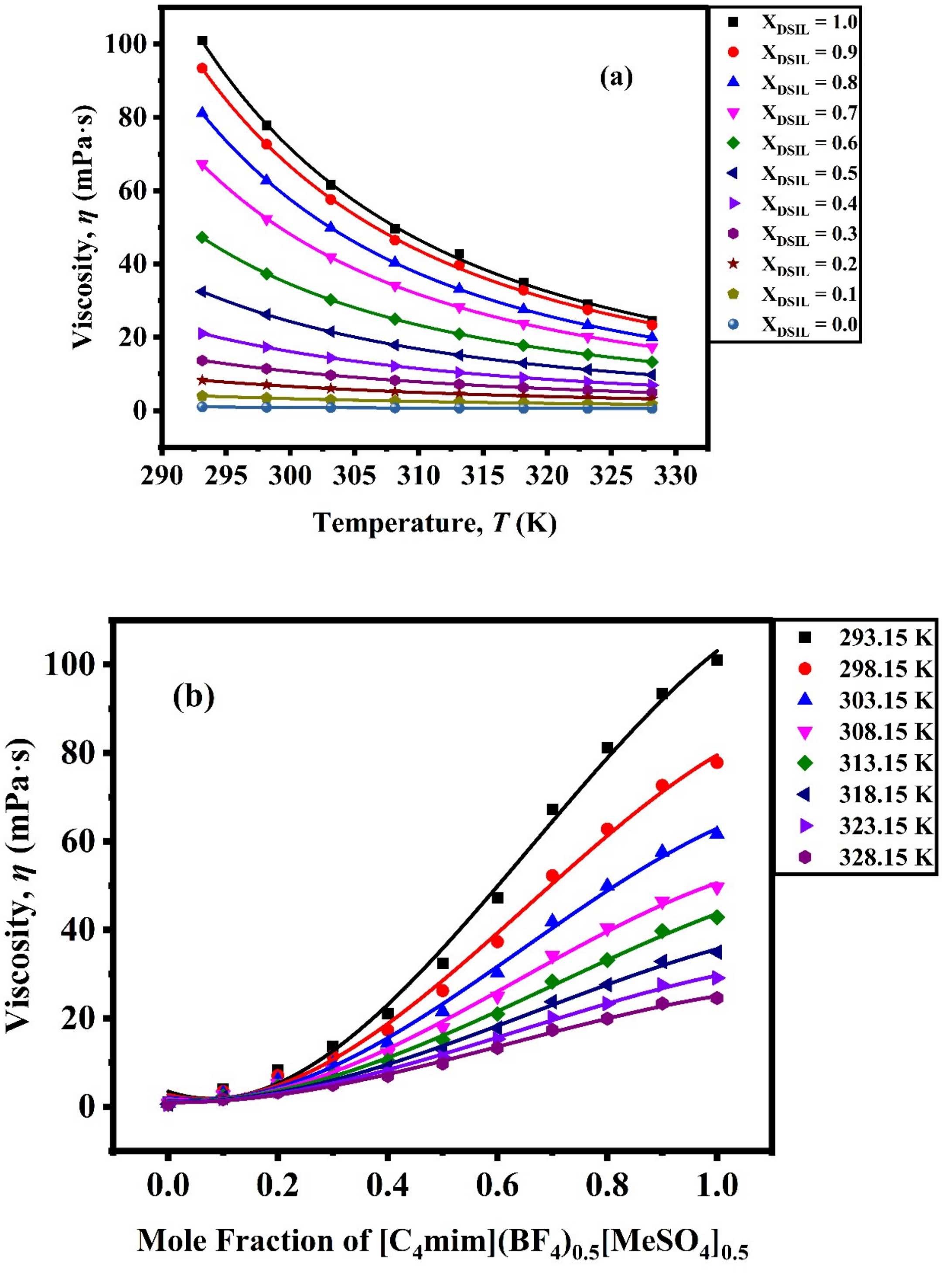

Fig. S18 Viscosity as a function of (a) temperature with VFT fitting and (b) mole fraction for $[C_4mim](BF_4)_{0.5}[MeSO_4]_{0.5}$-water mixtures.

Table S4. Vogel–Fulcher–Tammann (VFT) fitting parameters (A, B, and x_0) and corresponding coefficients of determination (R^2) obtained from fitting the temperature dependence of viscosity (η) for $[C_4mim](BF_4)_{0.5}[MeSO_4]_{0.5}$-water binary mixtures

X_{DSIL}	A	B	x_0	R^2
1.0	-0.317	232.191	193.095	0.999
0.9	-0.354	237.091	191.134	0.999
0.8	-0.576	267.942	185.340	1.000
0.7	-0.440	227.693	192.749	1.000
0.6	-0.411	204.108	195.242	1.000
0.5	-0.748	262.889	176.792	1.000
0.4	-0.776	245.892	176.043	1.000
0.3	-0.818	233.373	173.758	1.000
0.2	-1.041	257.268	161.799	1.000
0.1	-1.126	219.699	166.002	1.000
0.0	-0.762	49.574	228.851	0.992

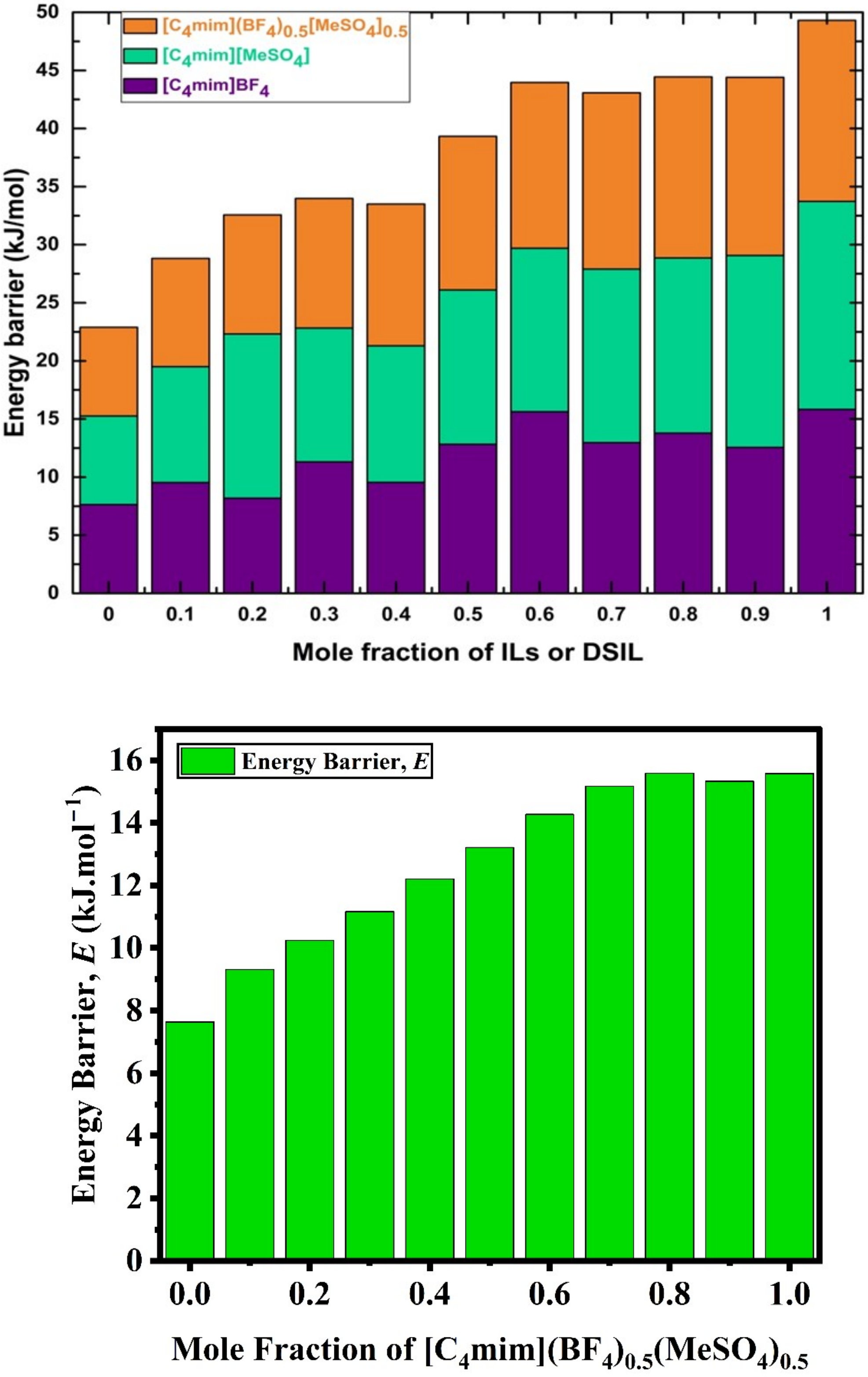

Fig. S19 Calculated energy barrier for DSIL and its component ILs.

Table S5. Experimental densities (ρ), dynamic viscosities (η), calculated V_m^E, partial molar volume ($V_{m,1}$), and viscosity deviation ($\Delta\eta$) for $[C_4mim](BF_4)_{0.5}[MeSO_4]_{0.5}$-water binary mixtures at different temperatures

x_{water}	ρ (g.cm^{-3})	V_m^E (cm^3.mol^{-1})	$V_{m,1}$ (cm^3.mol^{-1})	η (mPa.s)	$\Delta\eta$ (mPa.s)
T = 293 K					
0.0000	1.20771	0.00000	197.20710	101.0000	0.0000
0.1039	1.20742	-0.26917	197.06867	93.4100	2.4083
0.2064	1.20499	-0.26147	196.98117	81.1600	0.1567
0.3015	1.20238	-0.30471	196.91283	67.2500	-3.7550
0.4048	1.19826	-0.26843	196.81731	47.2500	-13.7566
0.4998	1.19290	-0.24403	196.66607	32.4700	-18.5383
0.6057	1.18447	-0.14963	196.90147	21.0400	-19.9700
0.7028	1.17409	-0.19203	197.06012	13.6900	-17.3216
0.7989	1.15521	-0.16250	196.60868	8.2800	-12.7333
0.9035	1.11592	-0.08305	196.39280	3.9930	-7.0219
1.0000	0.99871	0.00000	-	1.0166	0.0000
T = 298 K					
0.0000	1.20421	0.00000	197.78030	77.8500	0.0000
0.1039	1.20392	-0.26667	197.64105	72.6600	2.5046
0.2064	1.20149	-0.25484	197.55305	62.7900	0.3292
0.3015	1.19888	-0.29426	197.48115	52.2700	-2.4962
0.4048	1.19474	-0.25158	197.38122	37.3300	-9.7416
0.4998	1.18935	-0.22051	197.23244	26.2300	-13.1470

0.6057	1.1809	-0.12064	197.47129	17.3200	-14.3623
0.7028	1.17048	-0.15772	197.64253	11.4300	-12.5577
0.7989	1.15162	-0.12634	197.23512	7.0070	-9.2861
0.9035	1.11263	-0.05301	197.15876	3.4360	-5.1625
1.0000	0.99756	0.00000	-	0.9039	0.0000
T = 303 K					
0.0000	1.20072	0.00000	198.35510	61.6600	0.0000
0.1039	1.20043	-0.26461	198.21504	57.6000	2.0249
0.2064	1.19800	-0.24909	198.12531	49.9100	0.4198
0.3015	1.19538	-0.28391	198.04746	41.8700	-1.5352
0.4048	1.19120	-0.23327	197.95043	30.2300	-7.0903
0.4998	1.18580	-0.19821	197.80346	21.5700	-9.6654
0.6057	1.17731	-0.09190	198.04585	14.3800	-10.7705
0.7028	1.16687	-0.12576	198.23036	9.6720	-9.3936
0.7989	1.14801	-0.09219	197.85859	6.0200	-6.9606
0.9035	1.10928	-0.02468	197.91556	2.9890	-3.9067
1.0000	0.99616	0.00000	-	0.8108	0.0000
T = 308 K					
0.0000	1.19725	0.00000	198.93000	49.6400	0.0000
0.1039	1.19695	-0.26144	198.79064	46.4900	1.7407
0.2064	1.19453	-0.24415	198.69675	40.4100	0.5515
0.3015	1.19188	-0.27234	198.61940	34.1400	-0.8278
0.4048	1.18770	-0.21864	198.52056	25.0000	-5.0770
0.4998	1.18226	-0.17694	198.36441	17.8900	-7.2963

0.6057	1.17371	-0.06318	198.61626	12.1600	-8.1356
0.7028	1.16323	-0.09342	198.82300	8.2860	-7.1188
0.7989	1.14438	-0.05930	198.48666	5.2160	-5.2981
0.9035	1.10587	0.00259	198.66574	2.6230	-3.0003
1.0000	0.99454	0.00000	-	0.7326	0.0000
T = 313 K					
0.0000	1.19379	0.00000	199.50660	42.8600	0.0000
0.1039	1.19349	-0.26012	199.36574	39.7400	1.0993
0.2064	1.19106	-0.23852	199.27092	33.1800	-1.2414
0.3015	1.18840	-0.26296	199.19224	28.3000	-1.9020
0.4048	1.18420	-0.20425	199.08913	20.9700	-5.0127
0.4998	1.17872	-0.15635	198.93240	15.1200	-6.6434
0.6057	1.17013	-0.03711	199.19406	10.3800	-7.1641
0.7028	1.15962	-0.06454	199.40860	7.1730	-6.1518
0.7989	1.14073	-0.02755	199.10693	4.5620	-4.5434
0.9035	1.10242	0.02849	199.41112	2.3260	-2.5601
1.0000	0.99273	0.00000	-	0.6668	0.0000
T = 318 K					
0.0000	1.19036	0.00000	200.08150	34.9800	0.0000
0.1039	1.19005	-0.25766	199.94067	32.8700	1.3269
0.2064	1.18762	-0.23366	199.84491	27.5900	-0.5161
0.3015	1.18495	-0.25473	199.76012	23.7700	-0.8992
0.4048	1.18071	-0.18923	199.65435	17.8000	-3.4323
0.4998	1.17519	-0.13577	199.49808	12.9400	-4.8554

0.6057	1.16654	-0.01017	199.76377	8.9960	-5.3624
0.7028	1.15596	-0.03325	200.00016	6.2770	-4.6445
0.7989	1.13706	0.00357	199.73799	4.0230	-3.4616
0.9035	1.09892	0.05352	200.15108	2.0760	-1.9716
1.0000	0.99072	0.00000	-	0.6107	0.0000
T = 323 K					
0.0000	1.18692	0.00000	200.66140	29.1300	0.0000
0.1039	1.18662	-0.25862	200.51822	27.5400	1.2667
0.2064	1.18418	-0.23087	200.42066	23.3100	-0.1066
0.3015	1.18150	-0.24875	200.33123	20.1800	-0.3799
0.4048	1.17722	-0.17659	200.22281	15.3100	-2.3932
0.4998	1.17166	-0.11775	200.06698	11.1900	-3.6565
0.6057	1.16295	0.01408	200.34242	7.8540	-4.1357
0.7028	1.15233	-0.00673	200.5916	5.5480	-3.5850
0.7989	1.13338	0.03210	200.36058	3.5770	-2.6993
0.9035	1.09538	0.07650	200.88235	1.8680	-1.5516
1.0000	0.98850	0.00000	-	0.5629	0.0000
T = 328 K					
0.0000	1.18353	0.00000	201.23610	24.5900	0.0000
0.1039	1.18322	-0.25801	201.09155	23.3500	1.1622
0.2064	1.18076	-0.22692	200.99101	19.9200	0.1343
0.3015	1.17804	-0.23984	200.90263	17.3300	-0.0535
0.4048	1.17373	-0.16450	200.7969	13.2900	-1.6914
0.4998	1.16813	-0.10249	200.63901	9.7740	-2.8052

0.6057	1.15936	0.03334	200.91862	6.9390	-3.2380
0.7028	1.14867	0.01445	201.18499	4.9390	-2.8359
0.7989	1.12968	0.05207	200.99161	3.2050	-2.1677
0.9035	1.09181	0.08859	201.55901	1.6930	-1.2776
1.0000	0.98550	0.00000	-	0.5684	0.0000

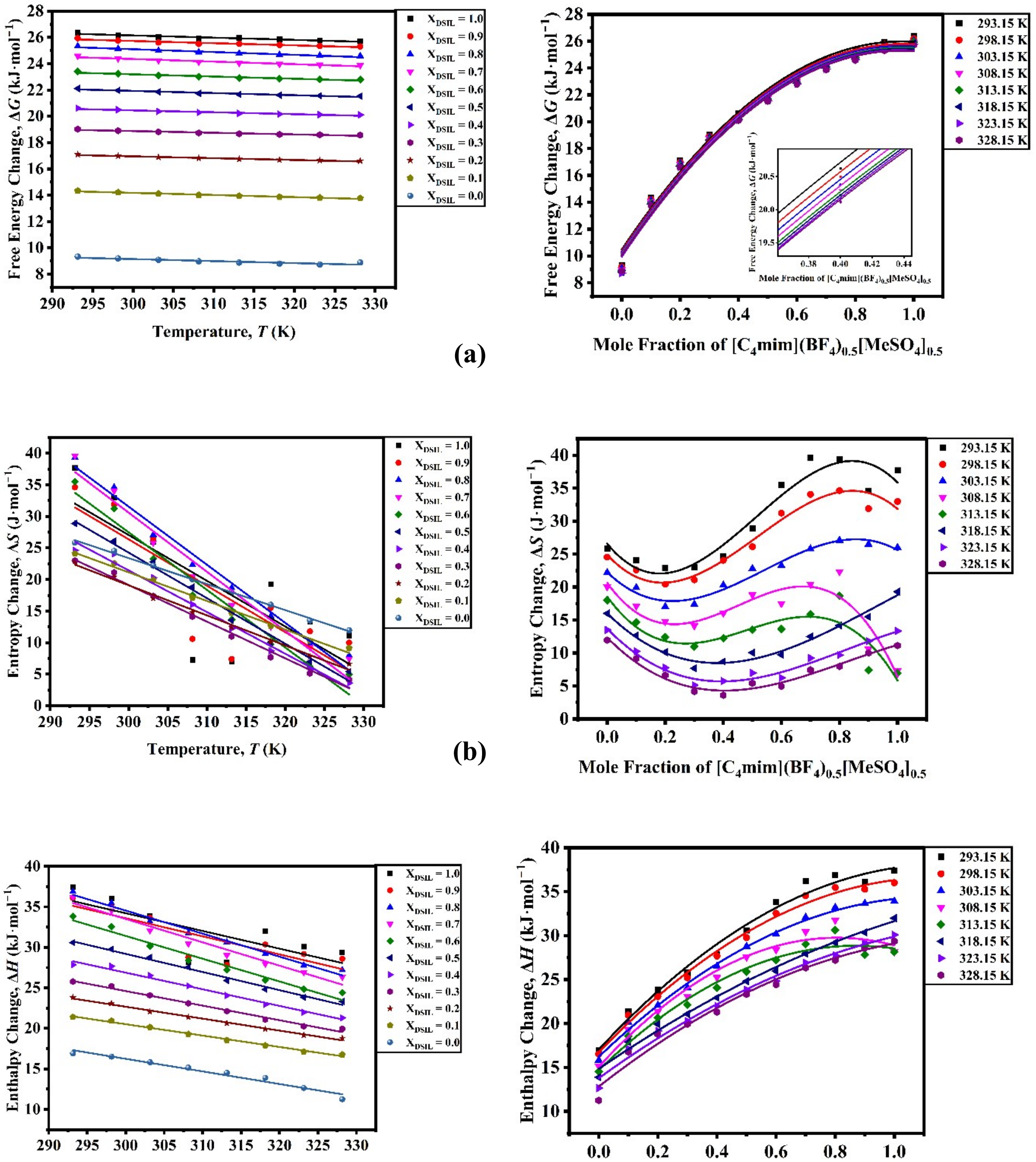

Fig. S20 (a) Free energy, (b) entropy, and (c) enthalpy changes of activation for viscous flow as a function of temperature and mole fraction for the binary mixtures of water and $[C_4mim](BF_4)_{0.5}[MeSO_4]_{0.5}$.

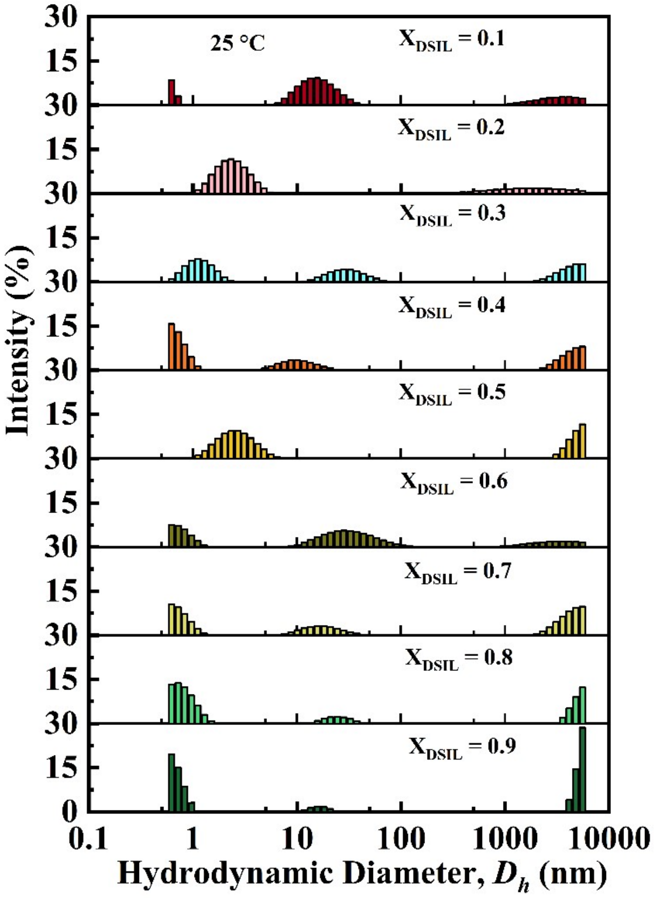

Fig. S21 Size distribution of aggregates formed in $[C_4mim](BF_4)_{0.5}[MeSO_4]_{0.5}$-water binary mixture from X_{DSIL} = 0.9 to 0.1 at 25 °C.

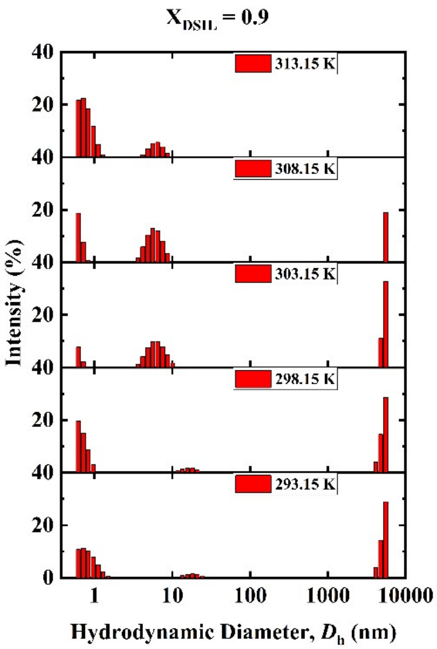
X$_{DSIL}$ = 0.9
313.15 K
308.15 K
303.15 K
298.15 K
293.15 K
Intensity (%)
Hydrodynamic Diameter, D_h (nm)

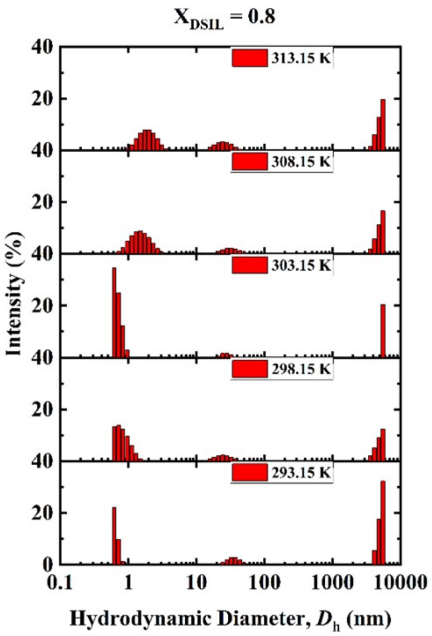
X$_{DSIL}$ = 0.8
313.15 K
308.15 K
303.15 K
298.15 K
293.15 K
Intensity (%)
Hydrodynamic Diameter, D_h (nm)

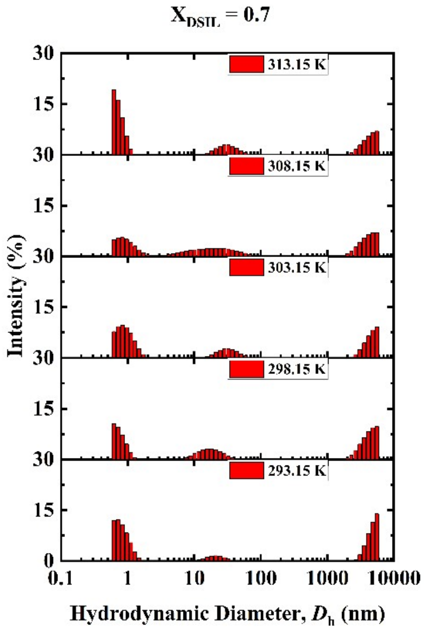
X$_{DSIL}$ = 0.7
313.15 K
308.15 K
303.15 K
298.15 K
293.15 K
Intensity (%)
Hydrodynamic Diameter, D_h (nm)

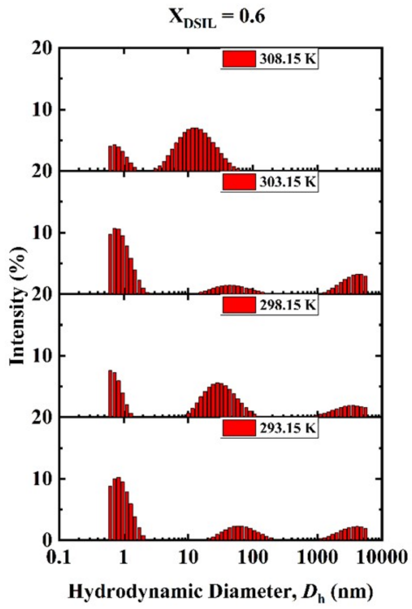
X$_{DSIL}$ = 0.6
308.15 K
303.15 K
298.15 K
293.15 K
Intensity (%)
Hydrodynamic Diameter, D_h (nm)

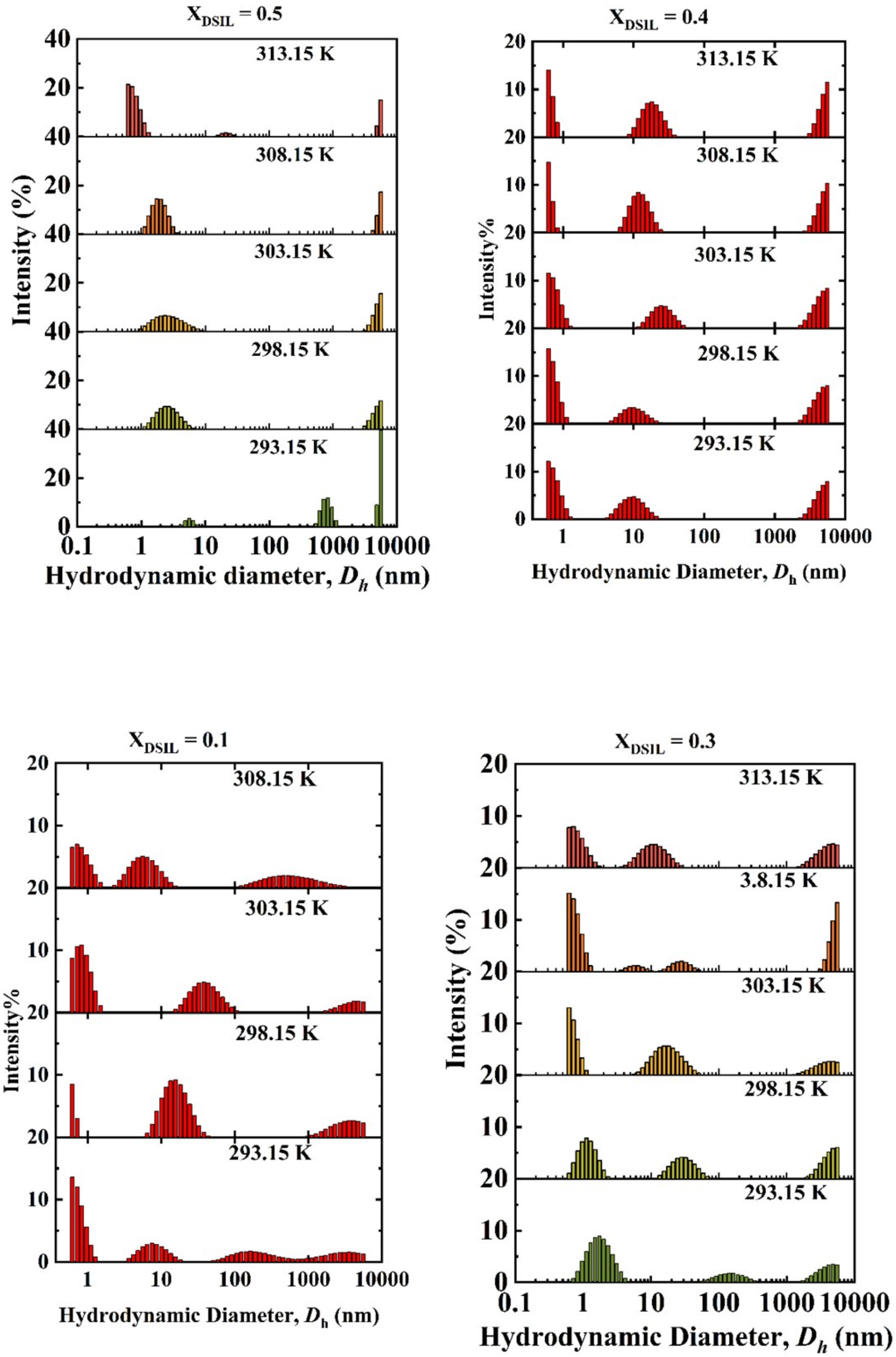

Fig. S22 Size distribution of aggregates formed in $[C_4mim](BF_4)_{0.5}[MeSO_4]_{0.5}$-water binary mixture for X_{DSIL} = 0.9, 0.8, 0.7, 0.6, 0.5, 0.4, 0.3, and 0.1 at several temperatures from 20 to 40 ℃.

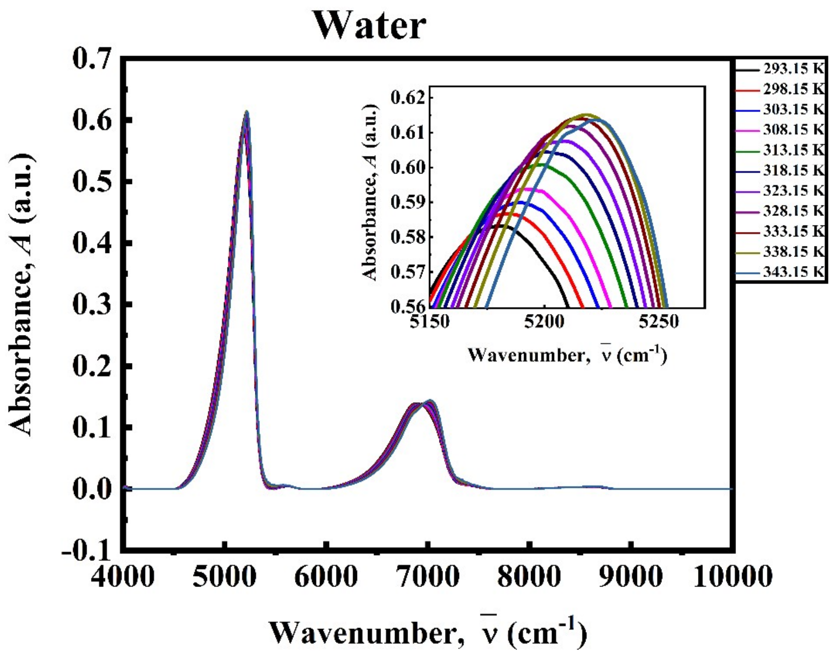

Fig. S23 NIR spectra of pure water from 20 to 70 °C.

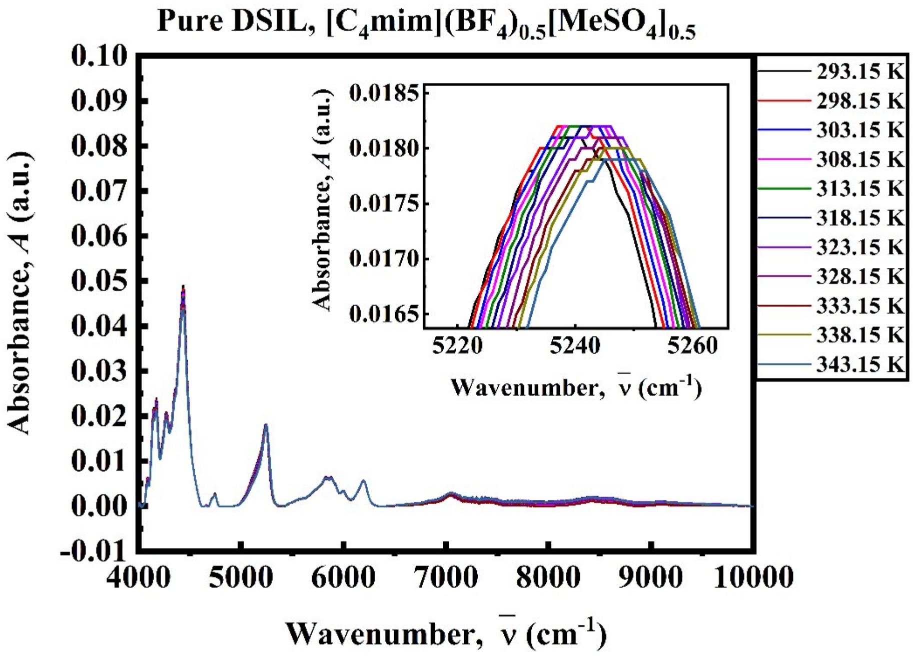

Fig. S24 NIR spectra of pure $[C_4mim](BF_4)_{0.5}[MeSO_4]_{0.5}$ from 20 to 70 °C.

Section S1

2D Correlation Analysis of Pure Water

The NIR spectra of water reveal that the absorption maxima rise progressively with increasing temperature, accompanied by a shift of the band toward higher frequencies. The amplification of band intensity upon increasing temperature arises from a higher concentration of free –OH groups in the equilibrium state. Moreover, the shift toward higher frequency suggests that the molecular vibrational modes require greater energy for excitation. This behavior arises because increasing temperature weakens intermolecular hydrogen bonds, thereby strengthening the covalent –OH bonds and causing their vibrations to occur at higher frequencies. To analyze the changing pattern of absorbance of different types of water species with temperature in detail, synchronous and asynchronous 2D correlation diagrams are generated from the dynamic spectrum of pure water. **Fig. S25** (a) and (b) exhibit the synchronous and asynchronous plots, respectively, of the 2D correlation analysis of the 4500-6000 cm^{-1} range.

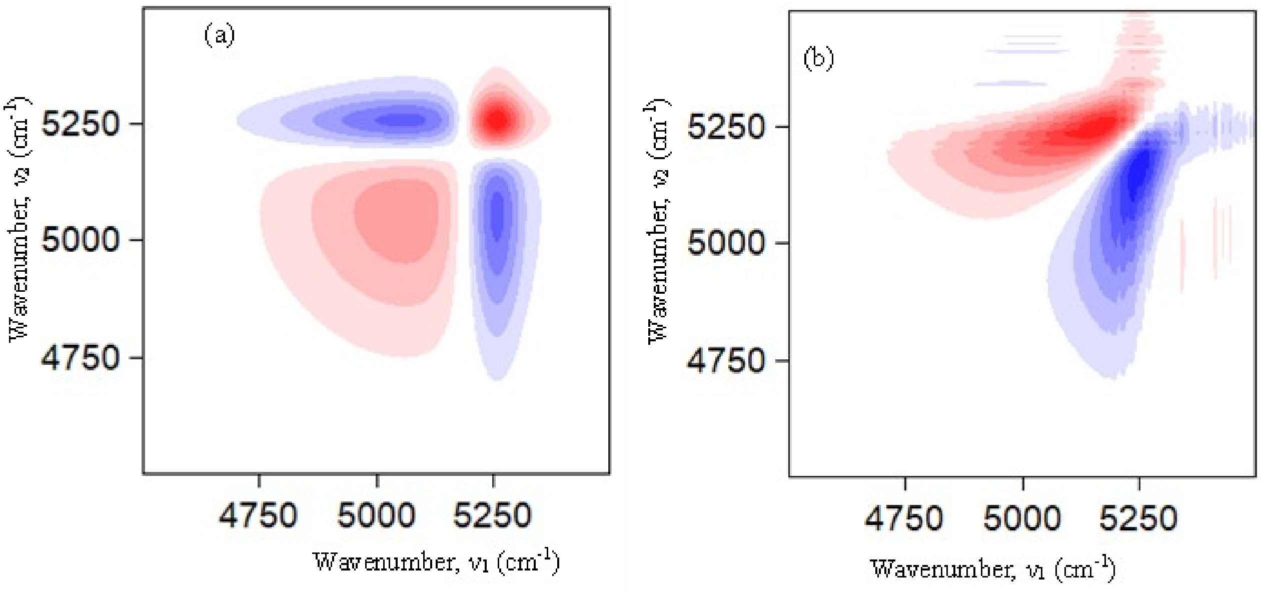

Fig. S25 (a) Synchronous and (b) asynchronous plot for pure water.

Autopeaks appearing at 5050 and 5254 cm^{-1} in the synchronous map indicate that the spectral changes at these positions are in phase. The dominant contribution to the declining intensity is observed near 5050 cm^{-1}, while the spectral peak at 5254 cm^{-1} strengthens progressively with increasing temperature. Negative cross-peaks between the autopeaks, together with their signs, suggest that the related spectral variations are negatively correlated. The asynchronous correlation spectrum exhibits maximum and minimum signals at 5254 and 5162 cm^{-1}, respectively, suggesting that the spectral variations are out of phase and that the transition at 5254 cm^{-1} occurs prior to or more quickly than at 5162 cm^{-1}. The synchronous plot shows that two principal spectral variations occur with increasing temperature, representing two different

components, with one transitioning into the other. This observation can be interpreted as a two-state model, where the two spectral positions, 5254 and 5050 cm^{-1}, reflect weaker and stronger H-bonded structures, respectively.

However, the peak observed at 5162 cm^{-1} in the asynchronous diagram indicates that a straightforward two-state model is insufficient to explain the structure of water. The band at 5162 cm^{-1} is a representative of a third intermediate state of water, which is less sensitive to temperature change compared to the other components. Therefore, the structure of water can be explained by a quasi-two-state model from this 2D correlation diagram. The stronger hydrogen bonds of the water species in the lower wavenumber region make the vibrations of the clusters of water, less sensitive to temperature change. Similarly, the weakly H-bonded water species corresponding to the band at 5254 cm^{-1} are more susceptible to temperature change.

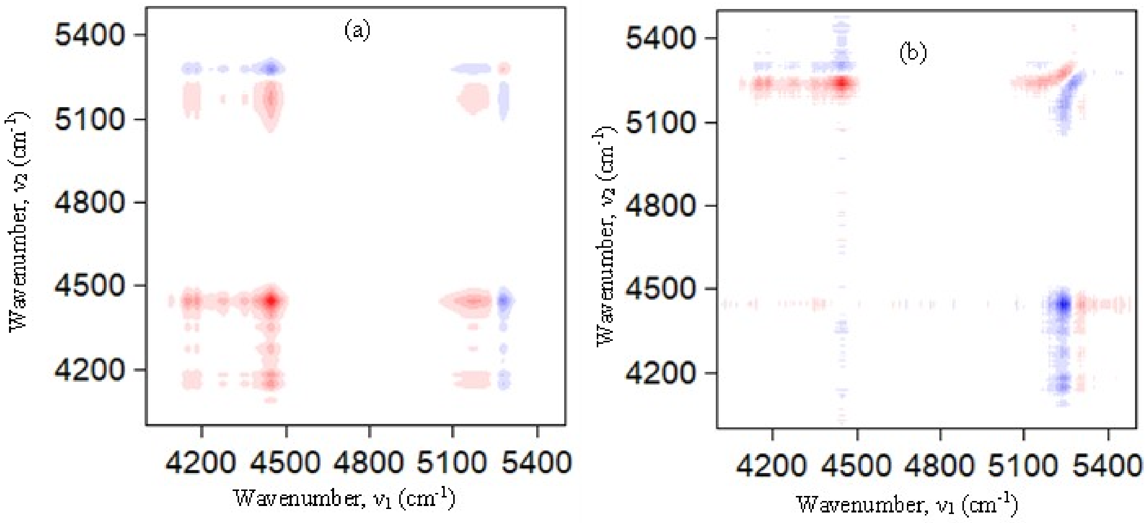

Fig. S26 (a) Synchronous and (b) asynchronous plot for pure $[C_4mim](BF_4)_{0.5}[MeSO_4]_{0.5}$.

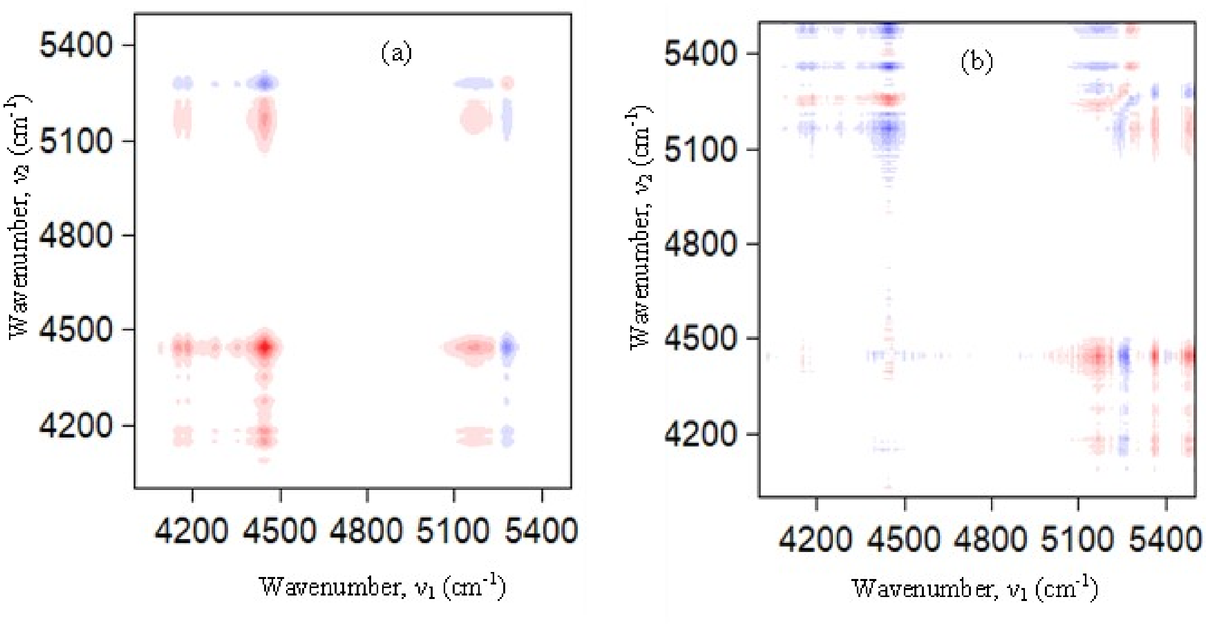

Fig. S27 (a) Synchronous and (b) asynchronous plot for 0.9 $[C_4mim](BF_4)_{0.5}[MeSO_4]_{0.5}$-water binary mixture.

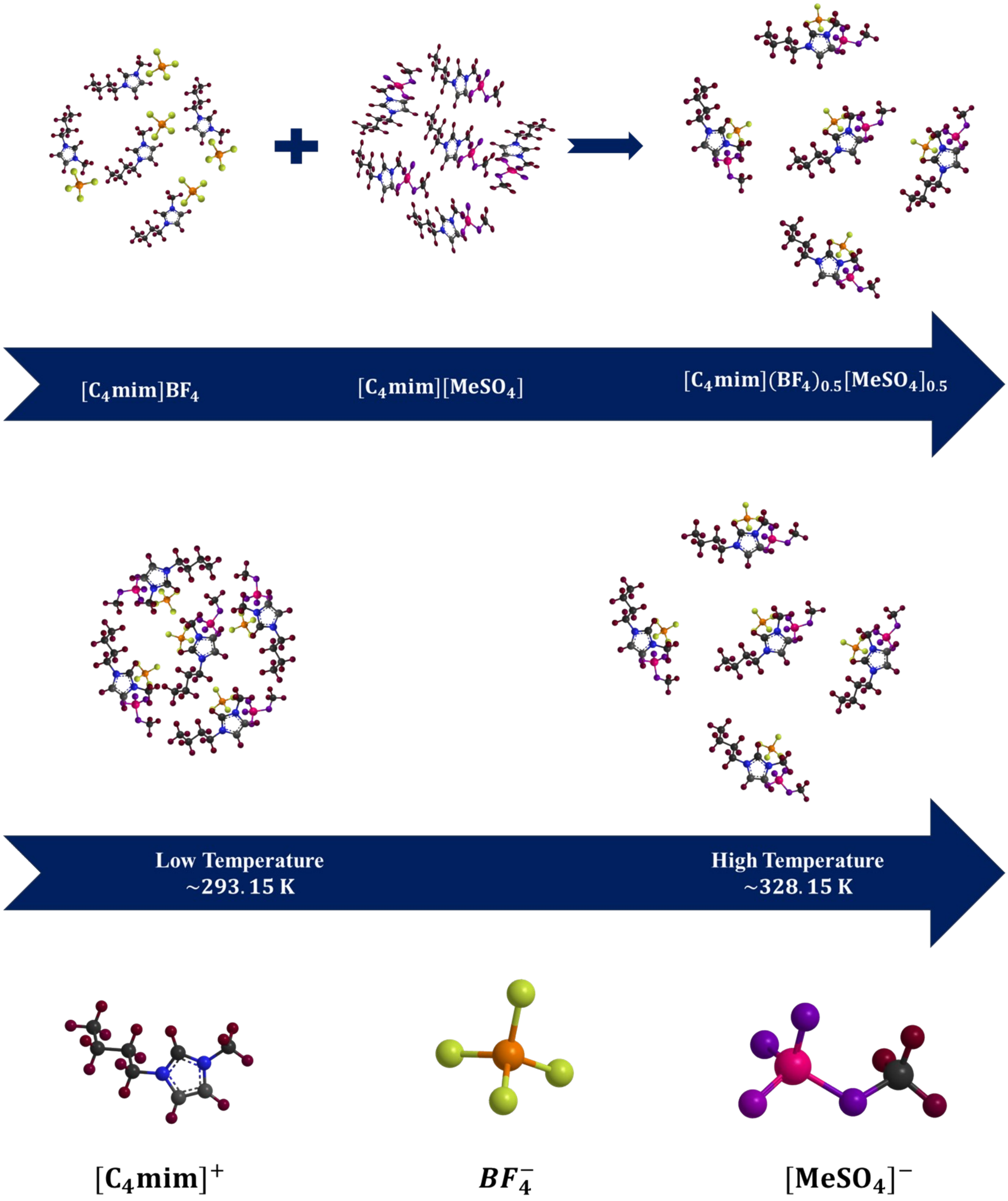

Fig. S28 Molecular-level interactions when (a) pure ILs are combined to form DSIL and (b) temperature increases gradually for DSIL.
